# Internalized SNCA/α-synuclein fibrils become truncated and resist degradation in neurons while glial cells rapidly degrade SNCA fibrils

**DOI:** 10.1080/15548627.2025.2579147

**Published:** 2025-11-12

**Authors:** Md. Razaul Karim, Elizabeth Tiegs, Emilie Gasparini, Riley Schlichte, Scott C. Vermilyea, Michael K. Lee

**Affiliations:** a Department of Neuroscience, University of Minnesota, Minneapolis, MN, USA; b Institute for Translational Neuroscience, University of Minnesota, Minneapolis, MN, USA; c Aligning Science Across Parkinson’s (ASAP) Collaborative Research Network, Chevy Chase, MD, USA

**Keywords:** Alpha-synuclein, endosome, lysosome, Parkinson disease, proteolysis, truncation

## Abstract

Parkinson disease (PD) and other α-synucleinopathies are characterized by the intracellular aggregates of SNCA/α-synuclein (synuclein, alpha) thought to spread via cell-to-cell transmission. To understand the contributions of various brain cells to the spreading of SNCA pathology, we examined the metabolism of SNCA aggregates in neuronal and glial cells. In neurons, while the full-length SNCA rapidly disappeared following SNCA pre-formed-fibril (PFF) uptake, truncated SNCA accumulated with a half-life of days rather than hours. Epitope mapping and fractionation studies indicate that SNCA fibrils internalized by neurons were truncated at the C-terminal region and remained insoluble. In contrast, microglia and astrocytes rapidly metabolized SNCA fibrils as the half-lives of SNCA fibrils in these glial cells were < 6 h. Differential uptake and processing of SNCA fibrils by neurons and glia was recapitulated in vivo where injection of fluorescently labeled SNCA fibrils initially accumulated in glial cells followed by rapid clearance while neurons stably accumulated SNCA fibrils at a slower rate. Immunolocalization and subcellular fractionation studies show that internalized SNCA PFF was initially localized to endosomes followed by lysosomes. The lysosome was largely responsible for the degradation of internalized SNCA PFF as the inhibition of lysosomal function led to the stabilization of SNCA in all cell types. Significantly, SNCA PFF causes lysosomal dysfunction in neurons. In summary, we show that neurons are inefficient in metabolizing internalized SNCA aggregates, partially because SNCA aggregates cause lysosomal dysfunction, potentially generating aggregation-prone truncated SNCA. In contrast, glial cells may protect neurons from SNCA aggregates by rapidly clearing these aggregates.

**Abbreviations**: 3MA, 3-methyladenine; aa, amino acids; AF, Alexa Fluor; Baf A1, bafilomycin A1; DMEM, Dulbecco’s modified Eagle’s medium; DMSO, dimethyl sulfoxide; FL, full-length; GAPDH, glyceraldehyde-3-phosphate dehydrogenase; HMM, high molecular mass; Hs, human; kDa, kilodalton; MAP1LC3/LC3, microtubule-associated protein 1 light chain 3; ML, molecular layer; NAC domain, non-amyloidal component; PCN, primary cortical neuron; PD, Parkinson diseases; PFF, pre-formed-fibril; PFF-488, PFF Alexa Fluor-488; PMG, primary microglia; SNCA, synuclein, alpha; SNCA[∆], C-terminally truncated SNCA; SQSTM1/p62, sequestosome 1; TX-100, Triton X-100.

## Introduction

Neurodegenerative diseases characterized by the presence of SNCA/α-synuclein (synuclein, alpha) aggregates are classified as α-synucleinopathies, including Parkinson disease (PD) and dementia with Lewy bodies (DLB). In PD and DLB, progressive neurodegeneration is accompanied by the presence of cytoplasmic SNCA aggregates [1]. SNCA is a highly conserved protein consisting of 140 amino acids that is predominantly expressed in neurons and enriched in presynaptic terminals. Under pathological conditions, SNCA adopts β-sheet conformations stabilized in oligomeric and/or fibrillar structures [2,3]. A series of studies now establish that SNCA pathology can spread from cell-to-cell [4–8].

While the mechanistic details of cell-to-cell spreading of SNCA pathology are still being uncovered, the general view is that a donor cell releases SNCA oligomer/aggregates, and the neighboring recipient cell internalizes the SNCA aggregates. Once in the recipient cell, internalized SNCA is thought to induce aggregation of SNCA in the recipient cell, possibly by acting as a seed for further aggregation. Although the details of how SNCA aggregates ultimately template or induce aggregation of endogenous SNCA are not fully understood, the metabolism/degradation of exogenous SNCA by recipient cells is an important factor in the spreading of SNCA pathology. In this regard, studies show that exogenous SNCA is taken up via endocytosis and trafficked via the endo-lysosomal pathway [9]. Presumably, the SNCA is eventually degraded via the major proteolytic pathways in the cell, including autophagy-lysosome and ubiquitin-proteasome systems [10,11].

In addition, studies show that different cell types in the brain may differentially metabolize internalized SNCA. For example, astrocytes can protect neurons from SNCA toxicity by competing for uptake of extracellular SNCA and degrading SNCA, presumably in the lysosome [6]. Similarly, microglia may coordinately degrade internalized SNCA via tunneling nanotubes and lysosomes [12,13]. However, these studies use cells that are exposed to relatively large amounts of SNCA aggregates for long periods. Thus, how different types of neural cells handle SNCA PFF immediately following uptake is not completely understood.

To better understand the contributions of various brain cell types in the spreading of SNCA pathology, we examined SNCA trafficking and metabolism at short time points following uptake in the major brain cell types (neurons, astrocytes, microglia, and oligodendrocytes). We show that microglia and astrocytes rapidly metabolize SNCA PFF where the half-lives of SNCA PFF in these glial cells are ~5 h. In neurons, while the full-length SNCA rapidly disappears following SNCA PFF uptake, substantial amount of C-terminally truncated SNCA stably accumulates and persists for days. We also confirm that internalized SNCA PFF is trafficked to endosomes followed by lysosomes. In glial cells, lysosome can completely degrade internalized SNCA. In neurons, while SNCA PFF is trafficked to lysosomes but is not fully degraded. Collectively, our results show that different brain cell types differentially metabolize internalized SNCA PFF and that glial cells could attenuate cell-to-cell transmission of α-synucleinopathy by reducing available SNCA aggregates.

## Results

### Exogenous SNCA fibrils accumulate as truncated species in neurons

While neurons internalize both exogenous SNCA monomers and fibrils, information regarding how neurons metabolize SNCA monomers and fibrils following the intracellular uptake is incomplete. We treated primary cultures of cortical neurons with Hu (human) SNCA monomers and PFF to study the kinetics of intracellular SNCA metabolism. To minimize the impact of continuous uptake of exogenous SNCA in the culture media in the analysis of SNCA metabolism, we removed exogenous SNCA after 2 h of uptake period. Briefly, the neurons were transiently exposed to exogenous SNCA for 2 h and then washed with phosphate-buffered saline (PBS) with and without trypsin-EDTA to remove any excess PFF attached to the outer membrane of neurons (Fig. S1). The amount of residual SNCA following PBS wash was comparable to the cells washed with PBS containing trypsin, which proteolyzed any residual SNCA attached to the outer membrane of neurons. Thus, the majority (~90%) of the remaining SNCA following the PBS wash represents internalized SNCA as they were resistant to the trypsin wash (Fig. S1 B).

**Figure 1.**
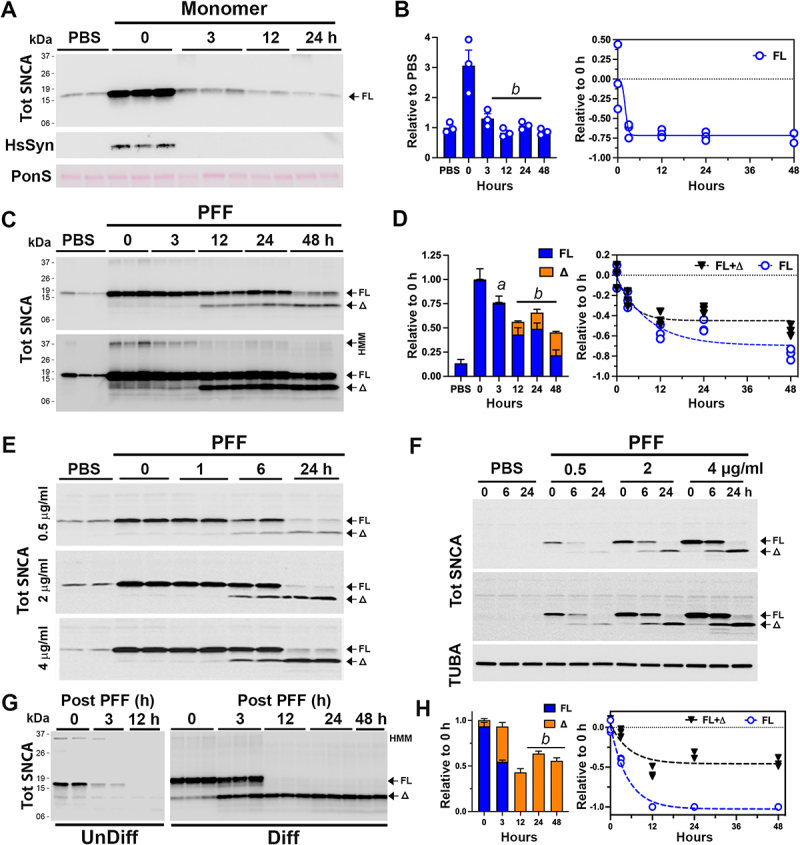
Exogenous SNCA PFF stably accumulates as a truncated variant in primary cortical neurons. Primary cortical neurons (PCN) cultured from newborn C57BL6/J mouse brains at 7 d in vitro (DIV) were used. PCN were pre-incubated with 4 µg/ml (unless otherwise indicated) (A, B) monomeric SNCA or (C-F) PFF for 2 h and washed to removed extracellular SNCA that was not internalized. Cells were provided with fresh media and then incubated for the indicated time before harvesting. (A) following SNCA monomer treatment, cell lysates were immunoblotted for total SNCA (tot SNCA) with Syn-1 antibody or HsSNCA using HsSyn antibody. Equal loading of proteins was verified using total protein stain (ponceau S, PonS). (B) quantitative analysis of tot SNCA immunoblot shows that internalized SNCA monomers are rapidly metabolized within 3 h of internalization. Mean±SEM; *n* = 3. *b, p < 0.001* vs 0-h, one-way ANOVA. (C) immunoblot analyses for tot SNCA following PFF treatment show that internalized SNCA[FL] is truncated (δ) and stably accumulate as the truncated species. Shown are two different exposure levels to show details. (D) quantitative analyses of tot SNCA immunoblot in (C). Bar and line graph show the kinetics of degradation and accumulation of SNCA species over time. Mean±SEM; *n* = 3. *a, p < 0.01*; *b, p < 0.001* vs. total SNCA (FL+Δ) at 0-h, one-way ANOVA. (E) to determine if the amount of SNCA PFF internalized affects the truncation and/or stability of internalized SNCA PFF, PCN was treated with 0.5, 2, and 4 μg/ml of SNCA PFF and the status of tot SNCA was analyzed at indicated times. Even at very low amounts of PFF (0.5 μg/ml), internalized PFF are rapidly truncated and stably accumulate. F) to determine if endogenous SNCA has an effect upon the metabolism of internalized SNCA PFF PCN established from *snca* KO mice were treated with SNCA PFF. The results show that the lack of endogenous SNCA does not affect the metabolism of internalized SNCA PFF. Shown are two different exposures for tot SNCA to show details. Equal loading of proteins was verified by immunoblotting for TUBA/α-tubulin. G) undifferentiated (UnDiff) or neuronally differentiated (Diff) hippocampal cell line (CLU198) were treated with 4 µg/ml SNCA PFF and internalized SNCA PFF were analyzed at indicated time. In UnDiff cells, internalized SNCA PFF is rapidly degraded by 12 h. In Diff cells, truncated SNCA (δ) stably accumulates. H) quantitative analysis of immunoblot shown in (G, Diff). The bar graph shows the relative status of full-length (FL) and truncated (δ) αS levels over time, confirming the stable accumulation of truncated SNCA. Mean±SEM; *n* = 3. *b, p < 0.001* vs total SNCA (FL+Δ) at 0 h, one-way ANOVA.

In neurons treated with SNCA monomers, the transient increase in total SNCA level rapidly decreases to the endogenous SNCA levels within 3 h (Figure 1(A,B)). Immunoblot analysis for the exogenous HsSNCA, using the HsSyn antibody [14], confirms the rapid loss of HsSNCA monomer in neurons (Figure 1(A)). In neurons treated with SNCA PFF, internalized SNCA persisted for a much longer period, with the approximate half-life of 12 h for the full-length SNCA (SNCA[FL]) (Figure 1(C,D)), indicating that the internalized SNCA PFF was more stable than the SNCA monomer. Significantly, SNCA PFF treatment led to the appearance of truncated SNCA (SNCA[Δ]) metabolites with a major species resolving at ~ 11 kilodalton (kDa) and corresponded to the disappearance of SNCA[FL] (Figure 1(C,D)). Significantly, SNCA[Δ] stably remained even after 48 h following uptake (Figure 1(C,D)). Finally, SNCA PFF treatment led to the stable accumulation of high molecular mass (HMM) SDS-resistant SNCA oligomers with the major Syn-1 antibody-reactive species resolving at ~ 37 kDa (Figure 1(C), lower panel). These results show that while neurons can rapidly degrade internalized SNCA monomers, internalized SNCA aggregates remain stable for a prolonged period. We note that “pulsing” SNCA PFF exposure by removing uninternalized SNCA PFF is important for accurate determination of SNCA PFF metabolism as neurons in unwashed cultures continued to accumulate more SNCA PFF over 48 h period (Fig. S1 D)

Because large amounts of internalized SNCA PFF may overload the cellular trafficking and degradation system, we asked whether the truncation and stability of internalized SNCA PFF depended on the amounts of internalized SNCA PFF. Cultured neurons were treated with 0.5, 2, or 4 μg/ml of SNCA PFF and the status of internalized SNCA was analyzed at various time following the PFF treatment (Figure 1(E)). The results show that even in neurons exposed to the lowest amount of SNCA PFF (0.5 μg/ml, ~30 ηM), internalized SNCA PFF stably accumulated as truncated species with similar kinetics to neurons treated with larger amounts of SNCA PFF (Figure 1(E)). Thus, generation and accumulation of SNCA[Δ] are intrinsic properties of SNCA PFF metabolism in neurons rather than a secondary effect of SNCA PFF overloading the protein degradation system. We also determined if endogenous SNCA expression affects the metabolism of exogenous SNCA PFF by treating cortical neurons lacking SNCA expression, established *from snca* knockout (KO) mice, with SNCA PFF (Figure 1(F)). Comparison with the wild-type (WT) mouse neuron (Figure 1(E)) showed that SNCA PFF is identically metabolized in both WT and *snca* KO neurons.

We previously showed that in neuronal cells, SNCA turnover slows with neuronal differentiation and maturation [15]. Thus, we examined whether neuronal differentiation has an impact upon the metabolism of internalized SNCA PFF. For this study, we used the mouse embryonic hippocampal cell line (CLU198) that can be induced to differentiate into neuronal phenotype [16] and can efficiently internalize SNCA PFF (Fig. S1 C). In undifferentiated CLU198 cells, internalized SNCA PFF, including the HMM species, disappeared rapidly (half-life of < 3 h) with no intermediate accumulation of SNCA[Δ] species (Figure 1(G)). In contrast, in neuronally differentiated CLU198 cells, the disappearance of SNCA[FL] was accompanied by the stable accumulation of HMM SNCA and SNCA[Δ] species (Figure 1(G)), even at 48 h following uptake (Figure 1(G,H)). Thus, the metabolism of SNCA PFF in neuronally differentiated CLU198 neuroblastoma cells was very similar to that seen in primary neurons (Figure 1(C-H)). Analysis of SNCA PFF metabolism in the SH-SY5Y Hu neuroblastoma cell line (Fig. S1 E) showed a similar pattern of SNCA metabolism as seen in CLU198 cells. Undifferentiated SH-SY5Y cells efficiently degraded SNCA PFF while in differentiated SH-SY5Y cells, internalized SNCA PFF stably accumulated as SNCA[Δ] (Fig. S1 E). Collectively, our results confirm that neuronal differentiation and maturation are associated with the distinct metabolism of SNCA PFF where the SNCA[FL] is rapidly processed and stably accumulates as SNCA[Δ].

### Internalized SNCA PFF is rapidly cleared by astrocytes and microglia but not oligodendrocytes

The differences in SNCA PFF metabolism between undifferentiated and neuronal differentiated states in the neuronal cell lines suggest that glial cells in the brain may be more efficient in metabolizing internalized SNCA PFF than neurons. Thus, we examined the clearance/metabolism of SNCA PFF in primary cultures of microglia, astrocytes, and oligodendrocytes. The purity of the cell types in cultures by immunohistochemical analysis for the cell type markers (Fig. S1 F-H). As with neurons (Fig. S1 B, C), analysis of cells washed with trypsin following 2 h exposure to SNCA PFF showed all cell types internalized SNCA PFF (Fig. S1 F-H).

**Figure 2.**
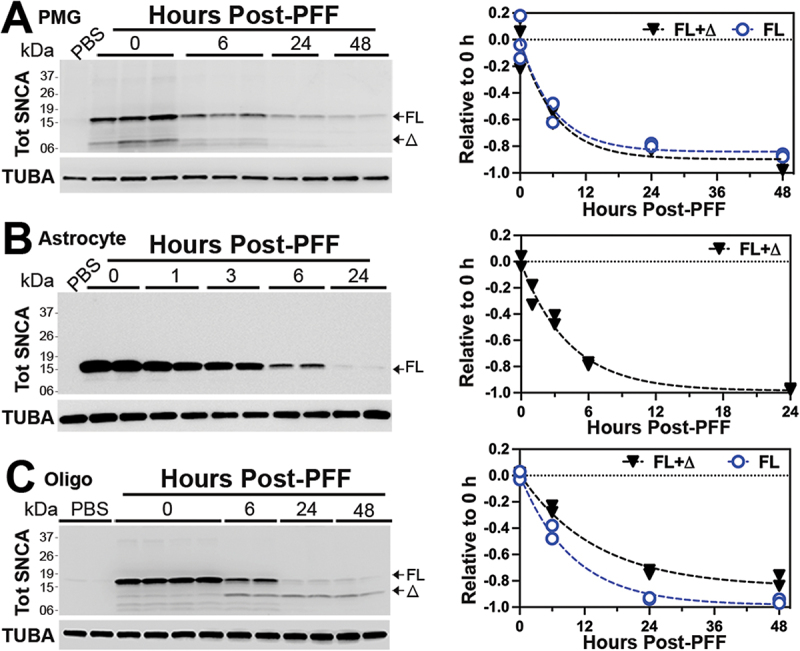
Glial cells rapidly degrade internalized SNCA PFF. (A) primary microglia (PMG), (B) primary astrocyte (astro), and (C) primary oligodendrocyte (oligo) cultures were established from newborn C57BL/6 mouse brains. Cells were pre-incubated for 2 h with 4 µg/ml SNCA PFF, harvested at indicated times post-washing, and immunoblotted for Tot SNCA. TUBA was used as a loading control. Quantitative analysis of the immunoblots (graphs) shows the rate of decrease in Tot SNCA. Note that most of the internalized SNCA PFF are degraded within 12–24 h with a much shorter half-life (~6 h) than in neurons. Further, astrocytes and microglia do not accumulate truncated SNCA seen in neurons. Significantly, oligodendrocytes stably accumulate truncated SNCA (δ), albeit at lower levels than in neurons. Mean±SEM; *n* = 2 per time point.

To define the clearance of internalized SNCA PFF, the cell lysates were collected at different times following the wash and analyzed for internalized SNCA. Our results show that both microglia and astrocytes efficiently metabolized SNCA PFF with the approximate half-lives of 6 h for microglia and 3 h for astrocytes (Figure 2(A,B)). Further, no obvious accumulation of SNCA[Δ] occurred in either of the cell types (Figure 2(A,B)). Analysis of Hu embryonic kidney cell line (HEK293) showed that, like the other non-neuronal cells, the HEK293 cells efficiently degraded the internalized SNCA PFF (Fig. S1 I, J).

Significantly, primary oligodendrocytes show distinct SNCA PFF metabolism compared to the other glial cell types. While the oligodendrocytes rapidly metabolized SNCA[FL], with a half-life of ~6 h, oligodendrocytes also generated SNCA[Δ] species that remained stable for a prolonged period (Figure 2(C)). Thus, the oligodendrocytes resemble neurons in the metabolism of SNCA PFF by generating and accumulating SNCA[Δ].

Thus far, our results show that microglia and astrocytes efficiently internalized and degraded SNCA PFF. However, neurons and oligodendrocytes did not fully metabolize SNCA PFF as SNCA[Δ] accumulated in these cells. While accumulation of SNCA[Δ] in SNCA PFF-treated neurons has been reported [17], this report is first to show that non-neuronal cells do not accumulate SNCA[Δ] and that oligodendrocytes also accumulate SNCA[Δ] following internalization of SNCA PFF. It is significant that both neurons and oligodendrocytes exhibit similar metabolism of internalized SNCA PFF as these are the two major cell types known to develop SNCA pathology in brain [18–20].

### Differential accumulation of SNCA PFF in neural cell types occur in vivo

**Figure 3.**
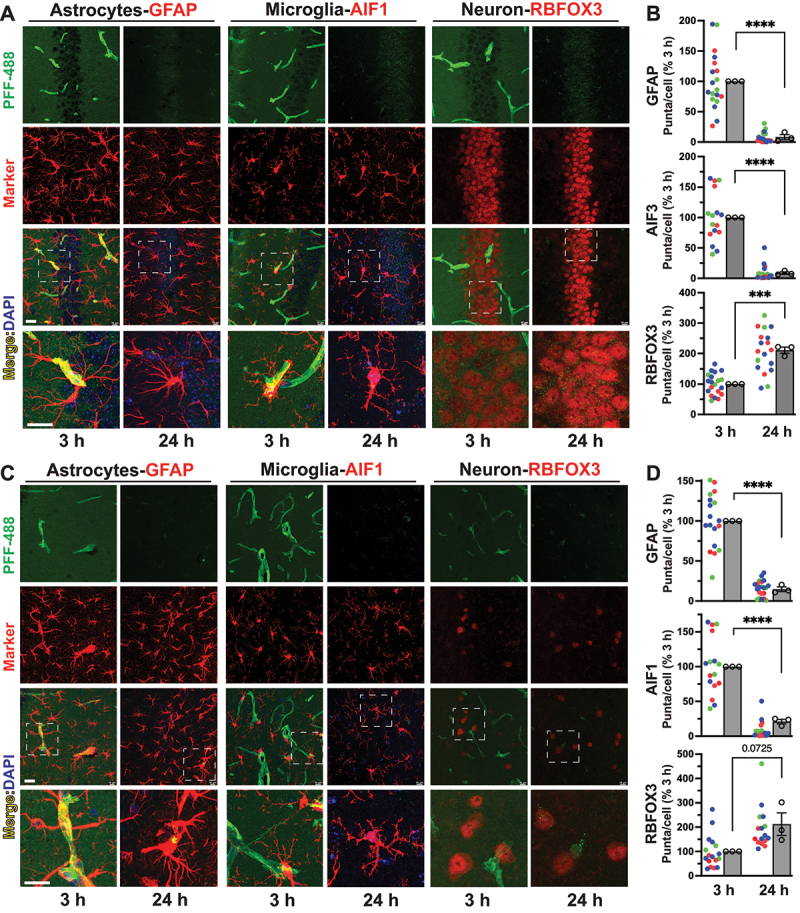
Exogenous SNCA PFF injected into brain is rapidly cleared by glial cells but accumulate in neurons. PFF-488 was stereotaxically injected into mouse brain (hippocampus/cortex) and the localization of PFF-488 was evaluated in brains harvested at 3 h and 24 h post injection. 20 µm thick free-floating section was immunostained with various cellular markers: astrocytes (GFAP); microglia (AIF1); neuron (RBFOX3) for CA1 (A, B) and molecular layer (ML)-dentate gyrus region (C, D) of hippocampus. Multiple confocal images were used to count the specific cellular colocalization of PFF using HALO quantitative image analyzer. The graphs are plotted as percentage (%) of 3 h average for each animal (B, D). Scatter plot represents an average value from a single section with the sections from same animal (3 animals per group, 3–8 sections per animal) plotted with same color (green, red, or blue). The bar graph shows the mean from each animal (3 animals per group) and compared using unpaired *t*-test. ****p < 0.001, ***p < 0.001, *n* = 3 per group, unpaired *t*-test, mean±SEM. Bar: 20 μm.

We examined if the exogenous SNCA internalization and accumulation is different between neurons and glia in brain. We injected SNCA PFF labeled with Alex Fluor (AF)-488 (SNCA PFF-488) into the CA2/3 area of mouse hippocampus (Fig. S2 A, B). The brains were collected at 3 h and 24 h post injection and processed for cellular localization of the SNCA PFF-488 (Fig. S2A, B). The cellular localization of the SNCA PFF-488 was examined at 2 different locations, CA1 pyramidal cell layer and ML (molecular layer) dorsal to dentate gyrus (Fig. S2 B). The sections were also immunostained to identify astrocytes (GFAP), microglia (AIF1/Iba1) and neurons (RBFOX3/NeuN) (Figure 3).

At 3 h post-injection, we observe prominent accumulations of SNCA PFF-488 along what appears to be vascular structures often lined with GFAP (Figure 3 (A,C)). The vascular accumulation is selective for the injected side as the SNCA PFF-488 signal is absent in the contra-lateral hippocampus (Fig. S2 C). Analysis of confocal slices indicate that the some of the vascular accumulation of SNCA PFF-488 occurs within GFAP processes suggest that the SNCA PFF-488 might be concentrating along the glial end-feet lining the glymphatic space (Fig. S2 D). Significantly, the vascular accumulation of SNCA PFF-488 disappears by 24 h post-injection (Figure 3(A,C)). In addition to the prominent vascular accumulation, we observe colocalization of punctate SNCA PFF-488 signals with glial cell markers (GFAP^+^ or AIF1^+^) at 3 h. This punctate SNCA PFF-488 within the glial cells are reduced at 24 h post injection (Figure 3(A-D)). We also observe a general green-fluorescence signal throughout the neuropil in the PFF injected side, but not in the contra-side, at 3 h that disappears by 24 h. We propose that this represents general spread of the injected PFF through the neuropil that is cleared by 24 h post injection (Figure 3 and Figure S2). In neurons, we observed a low level of SNCA PFF-488 colocalizing within the RBFOX3^+^ cells at 3 h post injection which increases significantly at 24 h post injection (Figure 3(A-D)). These results indicate that glial cells, particularly astrocytes, rapidly internalize exogenous SNCA fibrils and the internalized SNCA fibrils are efficiently cleared by the glial cells within 24 h. In neurons, while the accumulation of SNCA fibrils seem to occur slower, internalized SNCA fibrils persist past 24 h.

### Internalized SNCA PFF and truncated αS remain detergent insoluble in neuronal cells

When SNCA PFF are sonicated to facilitate uptake by the cells, sonication leads to a significant fraction of SNCA PFF partitioning into buffer soluble fractions that can induce seeding of SNCA aggregates [21]. Our analysis also confirmed that sonicated SNCA PFF used to induce SNCA pathology contained a significant amount of soluble SNCA (data not shown). Thus, we asked if the internalized SNCA are soluble or insoluble as they are metabolized by neurons. First, we compared the solubility of monomeric SNCA and SNCA PFF internalized by differentiated CLU198 cells (Fig. S3 A, B). CLU198 cells were treated with SNCA monomer (Fig. S3 A) or SNCA PFF (Fig. S3 B) for 2 h, washed, and the cells were collected at “0 h” and “24 h.” The collected samples were solubilized in Triton X-100 (TX-100) and the detergent soluble and insoluble fractions were obtained by centrifugation at 100,000xg. In the monomer-treated cells, the majority of SNCA remained soluble at 0 h and degraded by 24 h, albeit a small fraction of SNCA monomer, likely endogenous SNCA, was found in the insoluble fraction at 24 h. In the SNCA PFF-treated cells, the majority of αS species partitioned to the TX-100 insoluble fraction, even at 24 h. Significantly, TX-100 insoluble SDS-resistant SNCA oligomers of ~ 37 kDa seen at 0 h (Fig. S3 B, *) resolved at slightly lower MM at 24 h (Fig. S3 B, *Δ), consistent with the truncation of the HMM SNCA species.

Analysis of soluble and insoluble SNCA in the SNCA PFF-treated PCN during several days following PFF treatment show that the internalized SNCA remained insoluble at 0 d, 3 d, and 7 d following initial SNCA PFF internalization (Fig. S3 C). Consistent with very slow turnover of internalized SNCA PFF in neurons, ~50% of SNCA remained even at 7 d post internalization.

### Neurons accumulate C-terminally truncated SNCA PFF

To more accurately determine the size of the SNCA[Δ], we resolved SNCA PFF treated neuronal lysates next to the cell lysates containing SNCA proteins encoding aa (amino acids) 1–110(SNCA[110]), aa 1–120(SNCA[120]), aa 1–130(SNCA[130]), and FL [14] (Fig. S4 A). We observed that the major SNCA[Δ] species derived from SNCA PFF resolves between SNCA[110] and SNCA[120] with the estimated mass of ~11.5 kDa (Fig. S4 B).

**Figure 4.**
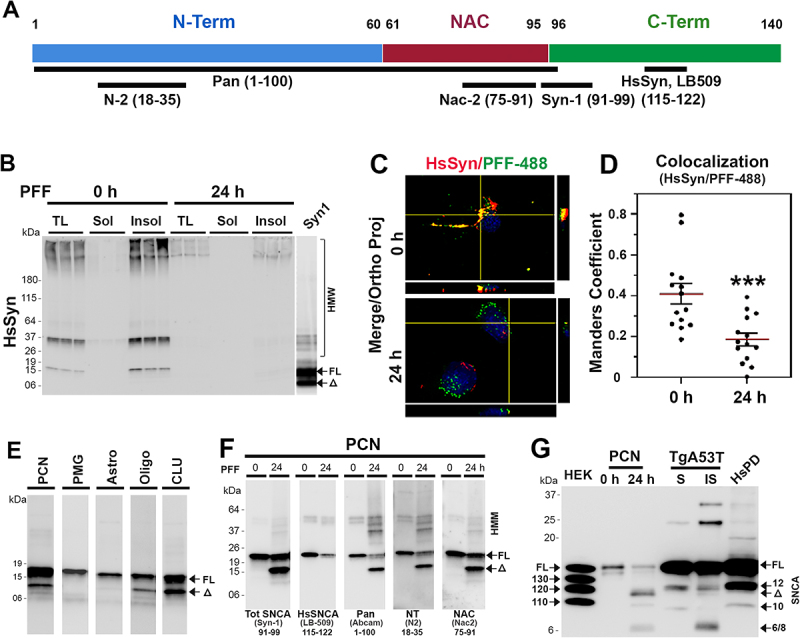
Neurons accumulate C-terminally truncated SNCA PFF. (A) schematic representation of αS with the locations of epitopes for the anti-SNCA antibodies used to map the truncated SNCA. (B) TX-100 soluble (sol) and insoluble (insol) fractions from PFF treated CLU198 cells were immunoblotted using HsSyn antibody. Note that HsSyn antibody recognizes SNCA[FL] but not SNCA[Δ]. (C) neuronally differentiated CLU198 cells were treated for 2 h with AF-488-labeled PFF and washed to remove any extracellular PFF-488. Cells were provided with fresh media and then incubated for 0 h or 24 h prior to fixing and immunostaining with HsSyn antibody. Double immunofluorescence microscopy was used to visualize HsSyn (red) and PFF-488 (green). (D) colocalization (yellow) between HsSyn signal and PFF-488 were quantified (*n* = 15–18 cells) by using the Manders’ coefficient (ImageJ software) and plotted. ***p < 0.001, unpaired *t*-test. (E) PFF treated PCN, PMG, primary astrocytes (astro), oligodendrocytes (oligo), and neuronal differentiated CLU198 cells (CLU) were analyzed for SNCA variants. All the samples were resolved on same gel but separated and reordered for clarity. Only PCN, oligo, and CLU accumulate SNCA[Δ]. (F) TX-100 insoluble fractions from PCN were immunoblot analyzed for tot SNCA (SYN-1, epitope 91–99), HsSNCA (LB509, epitope 115–122), N-terminal Pan-S (epitope 1–100), N-terminal NT-αS (epitope 18–35) and NAC-domain containing NAC-2αS (epitope 75–91). SNCA[Δ] is missing the C-terminal HsSNCA epitope but retains the N-terminal epitopes. (G) comparison of SNCA variants in SNCA PFF-treated PCN, PD case (HsPD) and TgA53T mice with SNCA pathology. In HsPD and TgA53T, SNCA [12] is the major truncated SNCA variant and SNCA[Δ] is a very minor component. In PCN treated with PFF, SNCA[Δ] is the dominant variant produced from internalized SNCA PFF.

We previously showed that SNCA is normally truncated at C-terminus and that C-terminally truncated SNCA is enriched in SNCA aggregates in vivo [14]. To determine if internalized SNCA PFF is C-terminally truncated in neurons, we performed immunoblot analysis of the soluble and insoluble fractions from PFF-treated CLU198 cells using the HsSyn antibody [14] that selectively recognize the C-terminal epitope (aa 115–122) on HsSNCA (Figure 4(A)). Our results show that the HsSyn antibody recognized both HMM and SNCA[FL] at 0 h but the HsSyn immunoreactivity was virtually absent at 24 h (Figure 4(B)). We also analyzed SNCA PFF-treated PCN at various times and compared the SNCA variants recognized by the Syn-1 and HsSyn antibodies (Fig. S3 D). As with the CLU198 cells, HsSyn-reactive bands disappeared with the loss of SNCA[FL], indicating that the 11.5 kDa SNCA[Δ] is missing the C-terminal portion of the protein. Taken together with immunoblot analysis of the SNCA species detected by Syn-1 antibody (Figure 1 and Figure S3), we conclude that the majority of internalized SNCA PFF was C-terminally truncated at 24 h.

To document the C-terminal truncation at the cellular level, neuronally differentiated CLU198 cells were treated with SNCA PFF labeled with Alex Fluor-488 (PFF AF-488, green) and immunostained with the HsSyn antibody and visualized using AF-647 (red) conjugated secondary antibody, at 0 h and 24 h following SNCA PFF treatment (Figure 4 (C,D)). Colocalization of PFF AF-488 (green) with the HsSyn immunoreactivity (red) showed high levels of colocalization between AF-488 and HsSyn immunoreactivity at 0 h. However, at 24 h following SNCA PFF treatment, colocalization of AF-488 with the HsSyn immunoreactivity dramatically decreased, indicating that the C-terminal portion of the SNCA was missing from the internalized PFF at 24 h (Figure 4(C,D)).

To further define the nature of the SNCA truncation, we used a variety of SNCA antibodies with the defined epitopes (Figure 4(A)) to map the primary structure of SNCA species generated by the cells following SNCA PFF internalization. Immunoblot analysis with Syn-1 antibody (amino acids 91–99) [14,22] (Figure 4(E)) showed that in PCN, CLU198, and oligodendrocytes, SNCA PFF treatment resulted in a major 11.5 kDa truncated SNCA[Δ], while the accumulation of truncated SNCA species were not obvious in PMG and astrocytes (Figure 4(E)). While small amount of SNCA[Δ] was occasionally seen in PMG, these species were transient as they disappeared with the SNCA[FL] (Figure 2(A)).

Because SNCA aggregates in vivo contain both *N*- and C-terminally truncated SNCA [14], we used additional anti-SNCA antibodies with defined epitopes located at the N-terminal region, C-terminal region, and central-NAC regions (Figure 4(A)) to map the SNCA[Δ] from the SNCA PFF-treated PCN (Figure 4(F)). The results show that the SNCA[FL] reacts with all antibodies. The major SNCA[Δ] reacted to antibodies recognizing the N-terminal (N2, Pan/pan-SNCA/SNCB) and the central (NAC, Syn-1) epitopes but not to the antibodies that bind to the C-terminal HsSNCA-specific epitope (LB509). The minor truncated variants at ~6–8 kDa only reacted to antibodies to central epitopes (Syn-1, NAC). Thus, internalized PFF was first truncated at the C-terminal region to generate ~11.5 kDa SNCA[Δ] and further truncated to remove the N-terminal region (SNCA[6/8]). Epitope mapping of SNCA species in primary oligodendrocytes (Fig. S4 C) and hippocampal CLU198 cell lines (Fig. S4 D) also show that these cells accumulated SNCA[Δ] where C-terminal epitope was missing while retaining the N-terminal and NAC regions. In PMG, a minor truncated species at ~ 6 kDa missing both *N*- and C-terminal region was observed (Fig. S4 E). Our results show that the stable accumulation of C-terminally truncated SNCA is a common feature of neuronal cells (PCN and CLU198 cells) and the oligodendrocytes, cell types that are associated with α-synucleinopathies.

To determine if the SNCA[Δ] seen in PFF treated neurons accumulate *in vivo* with SNCA pathology and to determine how the SNCA[Δ] compares to previously identified C-terminally truncated SNCA [14], we compared the SNCA from PFF treated neurons with the insoluble SNCA from the transgenic mouse expressing A53T mutant human SNCA (TgA53T) and human brain affected by α-synucleinopathy (Figure 4(G)). Insoluble aggregate recovered from TgA53T is qualitatively similar to the aggregates recovered from the PD cases [14] (Figure 4(G)) where the major C-terminally truncated species resolve at ~ 12 kDa (SNCA [12]). Comparison of brain lysates from TgA53T model and PD cases with the SNCA PFF treated neurons show that PFF-derived 11.5 kDa SNCA[Δ] (SNCA[11.5]) is qualitatively different from the insoluble SNCA aggregates recovered from the TgA53T mouse and PD case. Specifically, the major truncated species in TgA53T model and in human PD case resolved at ~ 12 kDa [14], while with the lysates from SNCA PFF-treated neurons, SNCA [12] was not seen and SNCA[11.5] was most abundant species (Figure 4(G)). We previously showed that insoluble SNCA from TgA53T model and PD case contains SNCA [12] as a major C-terminally truncated species, a minor C-terminal truncated species resolving ~10 kDa (SNCA [10]), and another truncated species at ~6–8 kDa lacking both *N*- and C-terminal regions (SNCA[6/8]) [14]. Thus, our comparative analysis shows that C-terminally truncated SNCA[11.5] is uniquely produced from SNCA PFF metabolism by neurons.

### Internalized SNCA PFF is trafficked to lysosomes via endosomes

Internalization of SNCA PFF occurs via the endosomal pathway and is trafficked to lysosomes [4,23–25]. Further, lysosomal proteases, such as cathepsins and asparagine endopeptidase (AEP), are implicated in the C-terminal truncation of SNCA [26–28]. Thus, we determined the time course of intracellular trafficking of SNCA PFF immediately after the internalization by treating the cells with SNCA PFF labeled with AF-488 or AF-647 (PFF-488 or PFF-647).

**Figure 5.**
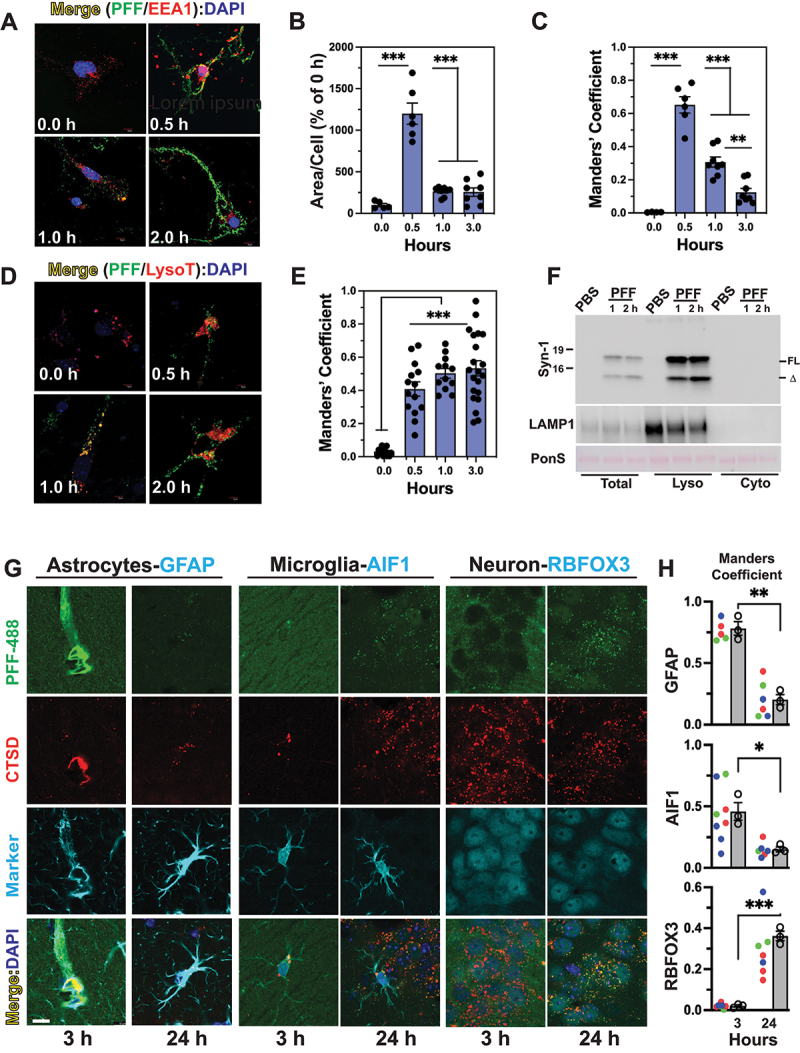
SNCA PFF is internalized via endosomes and trafficked to lysosomes. PCN was treated with PFF-488 and fixed at 0, 0.5, 1 and 3 h following PFF-488 addition. (A) cells were immunostained for early endosome marker EEA1. Both EEA1 (red) and PFF-488 (green) were imaged by double immunofluorescence microscopy. (B) quantitative analysis of the area/cell covered by EEA1 shows transient increase in early endosomes immediately following SNCA PFF uptake. (C) colocalization of EEA1 with PFF-488, expressed as the Manders’ coefficient, show initial colocalization of EEA1 and PFF-488 followed by a progressive decrease in colocalization. (D) PCN treated with PFF-488 were labeled with LysoTracker Red (LysoT). Both PFF-488 (green) and LysoT (red) were imaged using double immunofluorescence microscopy. (E) colocalization of PFF-488 with LysoT, expressed as the Manders’ coefficient, shows that PFF-488 is trafficked to lysosomes and continues to accumulate with lysosomes over time. (F) differentiated CLU-198 cells were treated with PFF for indicated time and cytosolic and lysosome-enriched fractions were obtained. Immunoblot analysis of the fractions shows that both SNCA[FL] and SNCA[Δ] partitions with the lysosome fraction. The fractions were also immunoblotted for LAMP1, a lysosomal marker. **p < 0.01, ***p < 0.001, one-way ANOVA. (G) lysosomal colocalization of internalized SNCA PFF in vivo. Brain sections from mice injected with PFF-488 (Fig. 3) was used to colocalize PFF-488 (green) with the lysosomal marker CTSD (cathepsin D [red]) and cell-type marker (astrocytes, GFAP; microglia, AIF1; neuron, RBFOX3). (H) colocalization of PFF-488 with CTSD in specific cell types are expressed as the Manders’ coefficient. Consistent with the internalization of pattern seen in Figure 3, SNCA PFF-488 colocalization with CTSD significantly increased in hippocampal neuron from 3 h to 24 h. In astrocytes and microglia, higher PFF-488 load appears at 3 h but significantly decreases at 24 h post injection. The scatter plot represents an average value from a single section with the sections from same animals (3 mice, 1–4 sections per mice) plotted with same color. The bar graphs show means for each animal (*n* = 3). *p < 0.05; **p < 0.01; ***p < 0.001, *n* = 3 per group, unpaired *t*-test, mean±SEM. Bar: 10 μm.

To confirm that internalized SNCA PFF in neurons are initially trafficked to endosomes, we colocalized internalized PFF-488 with an early endosome marker, EEA1, at different times following PFF-488 treatment (Figure 5(A-C) and Figure S5 A). At 0.5 h post SNCA PFF treatment, a significant increase in the amount of EEA1 staining was seen compared to the levels of EEA1 staining at other times (Figure 5(A,B) and Figure S5 A), indicating that internalization of SNCA PFF induced an increase in early endosomes. A strong colocalization of PFF-488 with EEA1 was seen at 0.5 h and the colocalization decreased at 1 h and 3 h post SNCA PFF treatment (Figure 5(A,C) and Figure S5 A). Following transit through endosomes, we hypothesize that a decrease in endosomal SNCA represents the trafficking of internalized SNCA PFF to lysosomes. To confirm this, we first treated PCN with the LysoTracker Red-50 followed by transient exposure to PFF-488. Colocalization of the PFF-488 with the LysoTracker was examined at 0, 0.5, 1, and 3 h post-PFF treatment (Figure 5(D,E) and Figure S5 B). Our results show that as the PFF-488 exits the endosomal compartment, PFF-488 accumulates in the lysosomes labeled by the LysoTracker (Figure 5(D,E) and Figure S5 B). Analysis of PMG and astrocytes (Fig. S5 C, D), show that internalized PFF-488 initially colocalizes with the endosome markers followed by the lysosome markers. We also performed subcellular fractionation to obtain LAMP1-enriched lysosomal fraction and cytosolic fraction from PFF-treated CLU198 cells (Figure 5(F)). Immunoblot analysis for SNCA showed that most of the SNCA, both SNCA[FL] and SNCA[Δ], is recovered with the lysosomal fractions.

Analysis of PFF-AF-488 with other subcellular markers in PCN show some colocalization of SNCA PFF-488 with LAMP2, SQSTM1, and endoplasmic reticulum marker, HSPA5/Grp78 (Fig. S5 E). No significant colocalization of internalized PFF-488 are seen with the markers of autophagosomes (MAP1LC3/LC3) or Golgi (GOLGA2/GM130) (Fig. S5 E).

To confirm that internalized SNCA PFF is also targeted to lysosomes in vivo, we imaged brain sections from the SNCA PFF-488 injected mice for colocalization of SNCA PFF-488 with lysosome (CTSD [cathepsin D]) (Figure 5(G)). We observed that in astrocytes and microglia, SNCA PFF-488 colocalizes with CTSD at 3 h post injection. In neurons, the low amount of SNCA PFF colocalized with CTSD at 3 h post injections but the overall number of SNCA PFF-488 puncta, as well as SNCA PFF-488 puncta colocalizing with CTSD increased at 24 h post injection. These results confirm that in vivo, exogenous SNCA fibrils are rapidly internalized glial cells and degraded by the lysosomes while the neurons exhibit a slower internalization of SNCA fibrils that accumulate within the lysosomes.

Collectively, our results support the scenario where internalized SNCA PFF traffic to lysosomes via the endosomes. Further, we proposed that the truncation of SNCA PFF (neurons) and/or degradation of SNCA PFF (all cells) occurs in lysosomes. In neuronal cells, internalized SNCA PFF stably accumulated in the lysosomes (Figure 5 and Figure S5) and the time course of SNCA PFF colocalization of lysosomes mirrors the timer course of SNCA[Δ] accumulation (see Figure 1 (C,E)).

### Lysosome is the major degradation machinery of SNCA PFF

Lysosome is an acidic organelle containing hydrolytic enzymes that are responsible for the degradation of protein aggregates, nonfunctioning intracellular organelles, and internalized foreign materials [10,29]. Given that the lysosome is the predominant destination of internalized SNCA PFF, we hypothesize that the lysosome is responsible for the rapid metabolism of SNCA PFF in non-neuronal cells. To directly test the role of lysosomes in SNCA PFF metabolism, we used Baf A1 (bafilomycin A_1_), which inhibits the V-ATPase responsible for the acidification of lysosomes requires the activation of resident hydrolytic enzymes [30].

**Figure 6.**
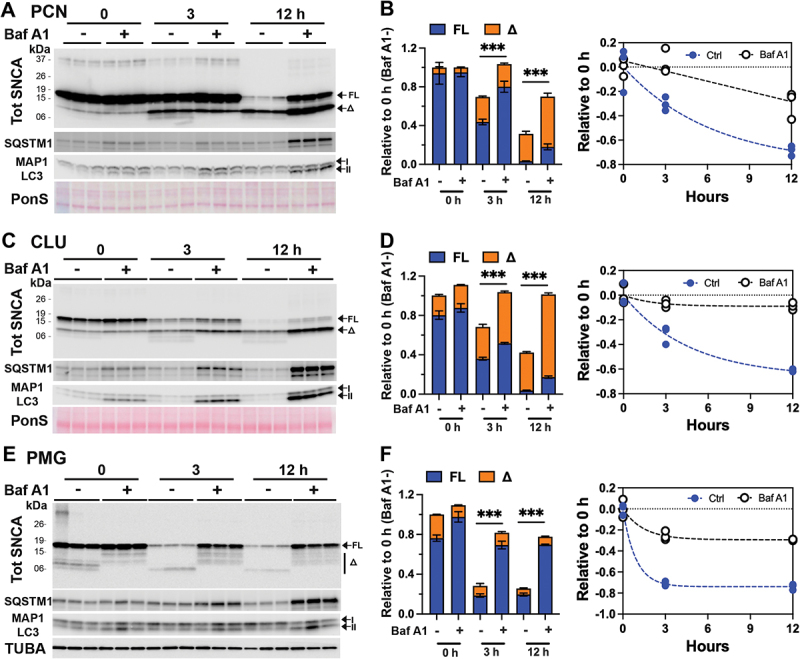
Lysosome function is required for the degradation of SNCA PFF in neuron and glial cells. Primary neuronal culture (PCN) (A, B), neuronally differentiated CLU198 cells (C, D), and primary microglia (PMG) (E, F) were pretreated with a lysosomal inhibitor bafilomycin A1 (100 ηM, Baf A1) or DMSO for 4 h. During the last 2 h of Baf A1 treatment, 4 µg/ml of SNCA PFF was added to the cells. The cells were washed and provided with fresh media containing DMSO (control, Ctrl) or Baf A1 (100 ηM) and harvested at 0, 3 and 12 h of incubation in fresh media. The levels of tot SNCA at each time point were determined by immunoblot analysis. Ponceau S (PonS) or TUBA was used as loading controls. Inhibition of lysosomes by BAF A1 treatment are indicated by increased accumulation of SQSTM1 and MAP1LC3-II in Baf A1 treated cultures. Bar graphs (B, D, F) show that levels of total SNCA (FL+Δ) in Baf A1-treated cells are significantly higher than in controls. Further, the individual levels of FL and SNCA[Δ] are significantly higher in Baf A1-treated cells at 12 h compared to the corresponding controls (*p < 0.001*, two-way ANOVA). The line graph and corresponding regression analysis show that Baf treatment significantly slows rate of SNCA degradation. Mean±SEM; *n* = 3. ***p < 0.001, total SNCA(FL+Δ) in Baf A1- vs Baf A1+, two-way ANOVA.

Baf A1 inhibition of lysosome in SNCA PFF-treated PCN resulted in stabilization of both SNCA[FL] and SNCA[Δ], particularly at 12 h post-PFF treatment (Figure 6(A,B)). In CLU-198 cells, Baf A1 also caused αS stabilization as in the PCN (Figure 6(C,D)). Because CLU198 cells exhibit a faster rate of SNCA PFF truncation, increased levels of both SNCA[FL] and SNCA[Δ] with Baf A1 treatment were obvious at 3 h following SNCA PFF uptake. Analysis of TX-100-soluble and -insoluble fractions showed that the Baf A1 treatment increased SNCA in the insoluble fraction (Fig. S6 A). Further, both monomeric and HMM SNCA were stabilized by Baf A1 treatment (Fig. S6 A).

We also treated non-neuronal cells with Baf A1 to test if lysosome function is responsible for the effective degradation of internalized SNCA PFF. Results from PMG (Figure 6(E,F)) show that Baf A1 treatment prevented the degradation of internalized SNCA PFF. Similarly, Baf A1 treatment also prevented degradation of SNCA PFF in primary astrocytes (Fig. S6 B) and HEK293 cells (Fig. S6 C).

These results show that in both neuronal and non-neuronal cells, lysosomal degradation is the predominant mode of degradation for internalized SNCA PFF. Significantly, in neurons, inhibition of lysosomes by Baf A1 does not inhibit SNCA truncation. Thus, while the truncated SNCA PFF accumulates in lysosomes, the truncation of SNCA PFF is independent of SNCA degradation.

Since the lysosome is largely responsible for the metabolism of internalized SNCA PFF in cells, we examined whether differences in the lysosomal content of the cells could be linked to differential metabolism of internalized SNCA PFF in the various cell types. When the cell types used here were analyzed for the abundance of lysosome markers (LAMP1 and CTSD) (Fig. S6 D, E), microglia and astrocytes exhibit higher levels of lysosomal markers than PCN, indicating that the glial cells have higher lysosomal capacity than in PCN. Moreover, undifferentiated CLU198 cells (CLU-UD; Fig. S6 D, E,) contain higher levels of lysosomal markers than the neuronally differentiated CLU198 cells (CLU-Diff; Fig. S6 D, E,). Query of single nuclei/cell RNAseq data bases (DropViz.org; brainrnaseq.org) also show that in adult mouse and Hu brain, LAMP1 and LAMP2 expression, representing lysosome content, is 2–3 fold higher in astrocytes/microglia than in neurons (Fig. S6 F). These results support the view that differences in the lysosomal activity contribute to the differential metabolism of internalized SNCA PFF in neural cell types.

**Figure 7.**
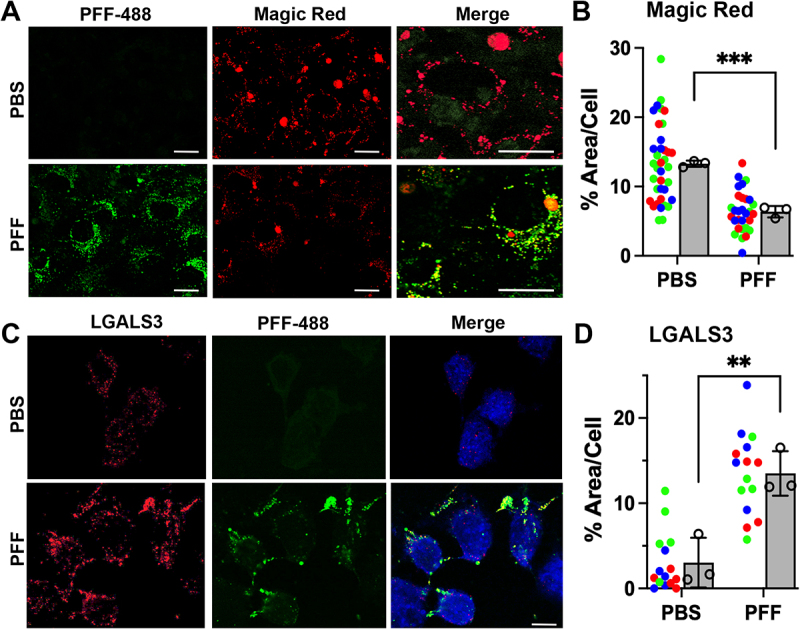
Internalized SNCA PFF inhibits lysosome function in neuronal cells. (A, B) neuronally differentiated CLU198 cells were treated with PFF AF-488 (PFF-488), washed, and incubated for 24 h prior to confocal live cell imaging. The cells were also treated with Magic Red CTSB assay reagent for the last hour prior to imaging. (A) Representative confocal live cell images of PFF-488 (green) and Magic Red (red). Merge shows higher magnification to show details. (B) scatter plot of % area/cell covered by Magic Red signal obtained from 3 independent cultures (8–16 cells per culture). Cells from the same replicates are shown in same color (green, red, or blue). The bar graph shows mean from each replicate. ****p < 0.0001, *n* = 3, unpaired *t*-test, mean±SEM. The overall Magic Red signal, representing CTSB activity, is significantly lower in PFF treated cells. (C, D) neuronally differentiated CLU198 cells were treated with PFF-488, washed, and incubated for 24-h, fixed in 4% PFA, and stained for LGALS3, an indicator of lysosomal damage. The cells were imaged using confocal microscopy. Representative confocal images show increased LGALS3 staining in PFF-treated cells (C). Percentage (%) of area positive for LGALS3 per cell, obtained from 3 independent cultures (5 cells per culture), is significantly higher in PFF treated cells (D). The scatter plots represent % LGALS3 area/cell with the cells from same replicate in same colors. The bar graph shows mean % LGALS3 area/cell for each replicate. ****p < 0.01, *n* = 3, unpaired *t*-test, mean±SEM. Bar:10 μm.

Accumulation of SNCA fibrils in the lysosome of neuronal cells is linked to dysfunctional lysosomes [4,10]. Thus, we tested if the accumulation of truncated SNCA PFF in lysosomes at early time points following PFF uptake was associated with lysosomal dysfunction. We focused on neuronal cells as the rapid degradation of SNCA PFF by glial cells excludes the possibility of lysosomal dysfunction by SNCA PFF in glial cells. Differentiated CLU198 cells were treated with saline or SNCA PFF-488 and at 6 h and 24 h post-SNCA PFF uptake, the lysosomal function was evaluated on live cells using the Magic Red CTSB activity kit (Figure 7(A) and Figure S7 A). Our results show that both 6 h and 24 h post-PFF uptake, the levels of CTSB activity were notably decreased in SNCA PFF-treated cells (Figure 7(A) and Figure S7 A). Quantitative analysis of Magic Red CTSB signal at 24 h following PFF-uptake showed that CTSB function, as indicated by the area occupied by the Magic Red signal per cell, was significantly decreased by SNCA PFF treatment (Figure 7(B)). Immunoblot analysis for CTSD in PCN and CLU cells treated with SNCA PFF show that SNCA PFF treatment leads to reduced levels of active CTSD relative to pro-CTSD (Fig. S7 B, C).

We also used LGALS3/galectin3 staining as a marker of lysosomal integrity [31]. LGALS3 is normally localized diffusely in the cytosol but when the lysosome becomes permeable/damaged, LGALS3 translocates into the lysosome and exhibits punctate staining. Analysis of cells treated with PBS or SNCA PFF shows that SNCA PFF treatments significantly increased the LGALS3 staining (Figure 7(C,D)). Finally, we analyzed lysosomal function in PCN using DQ-Red-BSA, which is targeted to lysosomes and resulting lysosomal proteolysis leads to an intense florescence signal [4]. In PCN, PFF treatment significantly decreased DQ-Red fluorescence (Fig. S7 D, F).

**Figure 8.**
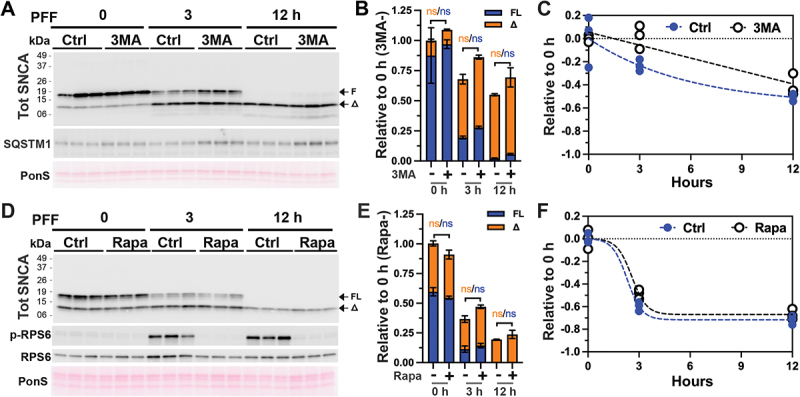
Autophagy is not a major factor in the metabolism of internalized SNCA PFF in neuronal cells. Differentiated hippocampal CLU198 cells and were pre-treated for 4 h with an autophagy inhibitor 3 methyladenine (100 mM, 3 MA) (A-C) or an autophagy activator rapamycin (100 ηM, rapa) (D-F). The cells were treated with 4 µg/ml SNCA PFF for the last 2 h of 3 MA treatment and washed. Washed cells were provided with fresh media containing PBS (Ctrl), 3 MA or rapa and then incubated for 0, 3, and 12 h before harvesting. Levels of tot SNCA were detected by immunoblot analysis (A, D). Also shown are the analysis of SQSTM1 (A), RPS6 (D), and p-RPS6 (D). Ponceau S (PonS) staining was used to verify equal protein loading. The bar (B, E) and line (C, F) graphs show the relative levels of full-length (FL) and truncated (∆) SNCA levels over time. Mean±SEM; *n* = 3. n.S., not significant.

We also evaluated the involvement of other possible proteolytic machinery in the metabolism of SNCA PFF. Given the important relationship between the lysosomes and autophagy, we examined the possible role of autophagy in the metabolism of SNCA PFF. We examined the role of autophagy on SNCA PFF metabolism by inhibiting autophagy via the 3 MA (3-methyladenine) treatment [32] (Figure 8(A-C) and Figure S8 A-D) and promoting autophagy via rapamycin treatment [33] (Figure 8(D-F) and Figure S8 E, F). 3 MA treatment of differentiated CLU198 cells led to modest increases in SNCA levels at 3 h and 12 h following SNCA PFF treatment but the increase was not statistically significant (Figure 8(A-C)). Non-neuronal cells treated with 3 MA showed no effect on SNCA levels in astrocytes (Fig. S8 A, B) and a modest increase in SNCA levels in HEK293 cells at 12 h following PFF treatment (Figure 8(C,D)). Stimulation of autophagy by rapamycin treatment did not lead to any obvious alternations in the levels of SNCA following PFF treatment of differentiated CLU198 cells (Figure 8(D-F)) and non-neuronal HEK293 cells (Fig. S8 F). Despite the lack of rapamycin on SNCA PFF metabolism, we observe that rapamycin treatment clearly inhibited MTOR (mechanistic target of rapamycin kinase) as indicated by the analysis of p-RPS6 and EIF4EBP1; and increased autophagy as indicated by the analysis of MAP1LC3 and SQSTM1 (Fig. S8E, F).

Analysis of the levels CTSD shows that 3 MA treatment decreases active CTSD levels in astrocytes and HEK293 cells (Fig. S8 B, D). Thus, we believe 3 MA treatment could be inhibiting the metabolism of SNCA PFF partly via induction of modest lysosomal deficit. Regardless, 3 MA affected SNCA PFF metabolism less than with the direct inhibition of lysosomes. Collectively, our results suggest that autophagy is a minor component in the regulation of SNCA PFF metabolism in neuronal cells. We also examined the potential role of the proteasome in SNCA PFF degradation in CLU198 and HEK-293 cells using, PS-341, a selective proteasomal inhibitor [34] (Fig. S8 G, H). We found that proteasome inhibition did not affect the metabolism of internalized SNCA PFF (Fig. S8 G, H). In conclusion, our studies indicate that lysosome is the major degradation machinery for the internalized SNCA PFF.

## Discussion

Progression of SNCA pathology in the brain is thought to involve cell-to-cell spreading of SNCA pathology where the pathogenic SNCA from doner neurons induces SNCA pathology in the neighboring neurons that internalize the pathogenic SNCA. In addition to neurons, glial cells can efficiently internalize extracellular SNCA variants released by neurons and may have an impact upon the spread of SNCA pathology. For example, astrocytes or microglia can attenuate the development of SNCA pathology in neurons by competing for the uptake of pathogenic SNCA [6,12,13,35]. However, most of the current studies examined the fate of exogenous SNCA at several hours or days following initial uptake. Thus, information about the short-term metabolism of the SNCA in various brain cell types is incomplete. To gain further insights about the roles of different brain cell types in the development of SNCA pathology, we examined how SNCA aggregates, in the form of SNCA PFF, are metabolized by various neural cells within hours of uptake rather than days. Our results show that neurons are inefficient in degrading internalized SNCA PFF and SNCA PFF stably accumulates as C-terminally truncated species. In neurons, we show that both the degradation and truncation of internalized SNCA PFF occurs in the endosome/lysosome compartment within minutes and hours following initial internalization of SNCA PFF. Moreover, truncation and stable accumulation of internalized SNCA PFF occurs in both primary cultured neurons as well as neuronal cell lines. Significantly, in neuronal cell lines (CLU-198 and SH-SY5Y), stable accumulation of truncated SNCA PFF is selectively associated with neuronal differentiation as undifferentiated cells rapidly degrade internalized SNCA PFF. Similarly, astrocytes and microglia rapidly degrade internalized SNCA PFF within 6–8 h while SNCA PFF accumulates as truncated species in oligodendrocytes. Epitope mapping of the internalized SNCA PFF shows that the major 11.5 kDa truncated species, SNCA[Δ], retains the N-terminal epitopes but presumably missing ~25 amino acids from the C-terminal region. We also show that the lysosome is a major organelle responsible for the degradation of internalized SNCA PFF as inhibition of lysosomes significantly increased accumulation of SNCA PFF in all cell types. Finally, we show that in vivo, exogenous SNCA fibrils injected into the hippocampus are rapidly taken up and cleared by the glial cells while neurons exhibit slower but longer-term accumulation of SNCA fibrils.

We originally showed that a significant fraction of SNCA is normally truncated at the C-terminal region and the C-terminally truncated SNCA can promote SNCA aggregation [14]. Moreover, the abundance of C-terminally truncated SNCA is increased in PD cases as well as in the SNCA aggregates in vivo [14]. However, the current report indicates that the truncation of internalized SNCA PFF is different than the truncation of SNCA expressed in cells or SNCA aggregates extracted from Hu and mouse cells/brain. While ~5–25% of endogenously expressed SNCA monomers accumulate as C-terminal truncated forms [14], internalized SNCA monomer is efficiently degraded without any accumulation of truncated SNCA. Further, SNCA aggregates extracted from mouse or Hu brains are partially truncated (~25–50%) [14], while virtually 100% of internalized SNCA PFF are truncated by neurons. Similarly, analysis of lysosomes isolated from the brains of a TgA53T mouse line shows that SNCA[FL] is more abundant than the truncated SNCA [28]. Direct comparison of truncated variants in SNCA PFF treated neurons with the SNCA aggregates extracted from the PD case and the TgA53T model show that, with the *in vivo* derived SNCA aggregates, the major C-terminally truncated species resolve at ~ 12 kDa [14], rather than ~11.5 kDa seen with SNCA PFF (Figure 4 G). While we have not defined actual site of SNCA truncation, other studies have shown that several lysosomal proteases can truncate SNCA at several C-terminal residues [28], including Glu114 and Asn103 [26,28,36]. Interestingly, cleavage at Glu114 is resistant to CASP inhibition [36], and cleavage at Asn103, while catalyzed by LGMN/asparagine endopeptidase, is independent of lysosomal pH [37]. Based on the size of the SNCA[Δ], it is unlikely that SNCA[Δ] is truncated at Asn103. While the report by Quintin and colleagues [36] did not examine differential metabolism of SNCA fibrils in different cells types, we believe it is likely that SNCA[Δ] observed in neurons is same as the SNCA truncated at Glu114 in HEK293 cells [36]. We do observe SNCA[Δ] in HEK cells treated with both SNCA PFF and lysosomal inhibitor (Fig. S6 C). Thus, we believe it is likely that very high levels of SNCA PFF (~14 ug/ml) used in the Quintin study [36] could be associated with lysosomal deficits. Overall, we provide a new observation that while astrocytes and microglia can rapidly degrade SNCA PFF without accumulation of SNCA[Δ], oligodendrocytes resemble neurons as both cell types accumulate truncated SNCA[Δ]. Thus, C-terminal truncation and accumulation of exogenous SNCA PFF is a common feature of the cell types that are known to develop SNCA aggregates in α-synucleinopathies.

A variety of other studies have shown that internalized SNCA or SNCA fibrils are targeted to lysosomes [4,23–25,38,39] and causes lysosomal deficits [4,9,23,40,41]. We have extended these studies by showing that internalized SNCA fibrils are differentially metabolized in neurons compared to non-neuronal cells. In neurons, we show that internalized SNCA fibrils are C-terminally truncated in endo-lysosomal compartment and stably accumulates. In contrast, glial cells rapidly degrade internalized SNCA fibrils. Further, we observe that similar kinetics of SNCA metabolism can be seen in vivo. Finally, we note that many of the prior studies on SNCA fibril metabolism utilize continuous treatment of the cells with very high levels of SNCA fibrils (0.7–1 μM, 8–15 μg/ml), which can artificially overwhelm the endo-lysosomal compartment, while we utilize much lower levels of SNCA fibrils (≤0.14 μM, ≤2 μg/ml).

Significantly, internalized SNCA PFF, even when extensively truncated, remains insoluble for at least 7 days, indicating that SNCA PFF remains aggregated in neurons for an extended period. Similar long-term stable accumulation of insoluble C-terminally truncated SNCA following PFF treatment was also observed with hippocampal slice culture [42]. The increased stability of the SNCA PFF in neurons may be because internalized SNCA PFF causes lysosomal dysfunction. However, this effect must not be a global lysosomal deficit but at an individual lysosome level since even very low levels of SNCA PFF are truncated and remain stable for an extended period. This result also indicates that even with the internalization of small amounts SNCA PFF by neurons, there is an extended timeframe for the internalized SNCA PFF to seed SNCA aggregation.

Collectively, we show that under normal conditions, any exogenous SNCA monomers are rapidly metabolized by all brain cell types but SNCA PFF, representing SNCA aggregates, are rapidly metabolized by glial cells but not by neurons. Our results suggest that studies on the cellular effects of SNCA PFF will need to consider the cell types used. We also predict that, if SNCA oligomers/aggregates are released extracellularly, astrocytes and microglia will efficiently remove the SNCA oligomers/aggregates under normal conditions, preventing significant transmission of SNCA oligomers/aggregates to neighboring neurons. However, under conditions that may lead to reduced SNCA uptake or lysosomal dysfunction, such as aging or increased inflammation, reduced metabolism of exogenous SNCA by glial cells likely promotes neuronal uptake of SNCA oligomers/aggregates and subsequent development of SNCA pathology.

## Materials and Methods

### Primary cell culture

Mouse pups between postnatal day 0–2 (P 0–2 d) were used to establish primary cultures of cortical neurons and glia were established as previously described [43,44] (For protocol see: . For *Primary Cortical Neuron (PCN)*, dissociated cells from newborn mouse cortex were plated onto a Matrigel (Corning Incorporated, 354,230)-coated cell culture dish (Corning Incorporated, 353,846) using (PM) plating medium (Dulbecco’s modified Eagle’s medium [DMEM; Thermo Fisher Scientific, 11,965,092], 1 mM sodium pyruvate [Thermo Fisher Scientific, 11,360–070], Glutamax [Thermo Fisher Scientific, 35,050–061], penicillin-streptomycin (Thermo Fisher Scientific, 15,140,122) and FBS (Thermo Fisher Scientific, A5256801) and on the following day, replaced with NbActiv4 (BrainBits LCC, NB4-500) containing FdU mitotic inhibitor (8 µM final; Sigma-Aldrich, F0503) to halt the growth of non-neuronal cells. Culture media was changed periodically twice a week until the cells became mature neurons [~12 d in vitro (DIV)], and then started various treatments as indicated. For *Mixed Glia culture*, 12,000,000 dissociated cells from the newborn brain were plated on a Matrigel-coated T75 flask using PM. Media was changed twice in a week until the cells became confluent (~7 DIV) (For glial isolation protocol see: *Primary Microglial cultures* were established from the confluent mixed glial culture in a T75 flask via shaking at 220 rpm, 37°C for 1 h. Floating microglia were pelleted by centrifugation (300×g for 5 min). The cells were resuspended in PM and filtered through a 70-µm cell strainer. Cells were plated for 48 h before the experiment. *Primary oligodendrocyte cultures* were established from the mixed glial culture depleted of microglia. Following microglial depletion, media was replaced and placed on a shaker (220 rpm, 37°C, overnight). Floating oligodendrocytes were filtered using a 40-µm cell strainer and spun down at 200×g for 10 min. Cells were resuspended in OPC medium (PM +50 µg/ml apo-TF [transferrin; Sigma-Aldrich, T1428], 5 µg/ml insulin [Sigma-Aldrich, 10,516], 30 ηM sodium selenite [MilliporeSigma, S9133], 10 ηM D-biotin [Sigma-Aldrich, B4639], 10 ηM hydrocortisone [Sigma-Aldrich, H0888], 20 ηg/ml PDGF-AA [Thermo Fisher Scientific, PHG0035] and 20 ηg/ml FGF2/bFGF [Thermo Fisher Scientific, PHG0264]). Cells were plated for 7–10 days before the experiment. *Primary Astrocyte cultures* were established following the removal of microglia and oligodendrocytes from the T75 flask. The remaining attached cells, representing astrocytes, were washed twice with PBS (Thermo Fisher Scientific, 10,010–023) and detached using 0.25% trypsin-EDTA, 5 ml NbAstro medium (Brain Bits LLC, NBAST) was added and filtered through a 40-µm cells strainer and spun down at 300×g for 10 min. The pellet was resuspended in NbAstro medium and filtered through 70- and then 40-µm cell strainers (Thermo Fisher Scientific, 22,363,548 and 22,363,547). Cells were counted and plated at 800,000 or 400,000 cells per well for 2–4 days before the experiment for biochemical or immunocytochemical analysis, respectively.

### Cell lines

CLU198 mouse hippocampal neuronal cell line , SH-SY5Y human neuroblastoma cell line , BV2 mouse microglia cell lines (Accegen Biotech, ABC-TC212S), and HEK-293 human embryonic kidney cell lines were used in this study. All cells were grown on full medium (DMEM, 10% FBS and 1% Pen-Strep). For differentiation of SH-SY5Y full medium was exchanged with differentiation media containing Neurobasal-A (Thermo Fisher Scientific, 10,888–022), pen-strep [Thermo Fisher Scientific, 15,140–122], B27 (Thermo Fisher Scientific, 17,504–044), and retinoic acid (Sigma-Aldrich, R2625). And for CLU the differentiation media contained Neurobasal-A, pen-strep, B27, and glutamax (Thermo Fisher Scientific, 35,050,061). Cells were differentiated for at least one week before being used for experiments.

#### Generation of recombinant SNCA/α-synuclein pre-formed fibril (PFF)

Recombinant wild-type human SNCA isolation, purification, and fibril formation were done as previously described [45] with modifications (For protocol see: [43]. Purified SNCA monomers were used to assemble SNCA PFF by agitation as previously described [43]. The PFF was diluted in PBS to 5 μg/μl aliquot and kept in a −80°C freezer. For internalization and processing study, PFF from frozen stock was diluted to 0.25 µg/µl in PBS, sonicated (1 s “on” then 1 s “off”) for 120 s with 20% amplitude by utilizing a Fisher Scientific Branson micro probe-tip sonicator (Fischer Scientific; Hampton, NH). Sonicated PFF were added into the media for the indicated time and doses (4 µg/ml, if otherwise indicated) to study primary cortical neurons, primary microglia, primary astrocytes, primary oligodendrocytes, hippocampal cell line CLU, SHSY5Y and HEK cells.

### SNCA preformed fibrils (PFF) uptake and degradation/clearance assays

For the uptake assay (For protocol see:, cells were treated with fresh cultured media containing 4 µg/ml of SNCA PFF and incubated for the indicated duration. Total lysates of fibril-treated cells were harvested at the end of the indicated time points.

For the clearance assaycells were pretreated with 4 µg/ml of SNCA PFF for 2 h, then washed with PBS or PBS supplemented with trypsin (0.005%) for 1 min to remove any excess PFF bound on the external cell surface. The washed cells were incubated with fresh media in the presence or absence of other drugs/inhibitors treatment. Cells were harvested at the indicated time points.

### Subcellular lysosomal fractionation

After the designated treatment, cells were washed thrice with DMEM and extracted in 0.5 ml of homogenization buffer (250 mM sucrose [Thermo Fisher Scientific, S5-3], 2 mM EDTA, 1.5 mM magnesium chloride, 10 mM potassium chloride, and 20 mM HEPES, pH 7.4) supplemented with proteinase (Sigma-Aldrich, 11,836,170,001) and phosphatase (Sigma-Aldrich, P5726 and P0044) inhibitors . The cells were gently detached using a cell scraper and homogenized using a Teflon homogenizer (DWK Life Sciences, Part No. 358,005; 12 strokes). Fifty µl of total homogenates (TH) were separated and lysed with TNE lysis buffer (50 mM Tris, 150 mM NaCl, 5 mM EDTA adjusted to pH at 7.4). The rest of the TH was centrifuged at 1000×g for 10 min at 4°C. The resulting supernatant was centrifuged for 20,000×g for 20 min at 4°C to collect the precipitate as a crude lysosomal fraction (CLF). The CLF was lysed with TNE lysis buffer and used for western blot analysis.

#### TX-100 fractionation of soluble and insoluble SNCA

Protein extraction and fractionation into total lysates (TL), TX-100 (Sigma-Aldrich, T8787) soluble (S), and insoluble (IS) fraction was conducted as described previously [14, 43]. Briefly, cells were washed using cold PBS, TNE +1% TX-100 was added on ice for 5 min and sonicated to achieve the TL fractions. The TL was centrifuged using an Airfuge (Beckman Coulter) at 100,000xg for 10 min. The supernatant was adjusted to complete TNE lysis buffer and considered as a soluble fraction. Washed pellets were re-suspended in complete TNE lysis buffer as the insoluble fractions.

#### Protein extraction and immunoblot analysis of protein expression

Cells were lysed in TNE lysis buffer containing 1% SDS (Sigma-Aldrich, L4509), 0.5% NP-40 (Sigma-Aldrich, 74,385), 0.5% DOC [10]. Immunoblot analysis was conducted as described previously [10,43,46]. Briefly, relative protein levels of total SNCA (Syn-1), Hs-specific SNCA (HsSyn or LB509), SNCA/NAC-2, SNCA/N-2, SQSTM1, LC3 and other proteins were determined from cell extracts by quantitative immunoblots analysis using chemiluminescence detection of horseradish peroxidase-conjugated secondary antibodies on the GE Image-Quant LAS-4010 (GE Healthcare, Waukesha, WI). Image-Quant software (GE; RRID:SCR_014246; https://www.cytivalifesciences.com) was used to determine the intensity of the immunoreactive bands [10,43,46].

#### Labeling of SNCA pre-formed fibril with Alex-Fluor 488 (PFF-488)

Alexa Fluor (AF) 488 Microscale Protein Labelling Kit (Invitrogen, A30006) was used to label PFF according to the manufacturer’s protocol . AF 488 reactive dye has a tetrafluorophenyl (TFP) ester moiety that is more stable in solution than the commonly used succinimidyl (NHS) ester. TFP ester reacts efficiently with primary amines of protein to form a stable dye-protein conjugate and is independent of pH between 4 and 10. Hs-WT-SNCA PFF were diluted at a concentration of 1 µg/µL in PBS followed by sonication (1 s on then 1 s off) for 120 Sec with 20% amplitude. Sonicated PFF were used to label with AF 488.

#### Immunocytochemistry

Immunocytochemistry was conducted as described previously [10,43,44] . Briefly, cells grown on coverslips were fixed in 4% paraformaldehyde (PFA) and immunostained followed by confocal imaging. Alex fluor (AF-647 or AF-488) conjugated secondary antibody was used.

#### Analysis of SNCA PFF-488 uptake in vivo following intrahippocampal injections of SNCA PFF-488

Alexa Fluor 488-conjugated SNCA PFF (PFF-488) described above were injected into to covalently label 5-month-old SNCA knockout mice (*snca*^−/−^; *n* = 3 per timepoint). The mice were anesthetized using 3% isoflurane mixed with oxygen, placed on a stereotaxic frame, and the mice received a unilateral injection of 2.5 μL total volume per depth (185 ng/µl)) using a 10 μL syringe (Hamilton; Reno, NV, USA) at a rate of 200 nL/min into the hippocampus and overlying cortex (coordinates: +2.5 mm mediolateral; −2.4 mm anteroposterior; −2.4 mm followed by −1.0 dorsoventral from the skull). Injections were followed by a 5 min resting period prior to removal of the needle. The brains were collected following 3 h and 24 h post injection.

For brain collection, the mice were anaesthetized with isoflurane and intracardially perfused with potassium-free PBS (Thermo Fisher Scientific, J60465.AP) followed by 4% PFA in PBS. Brains were removed and postfixed in PFA for 24 h, cryoprected in 30% sucrose solution, and 20 µm thick free-floating coronal sections were obtained using a freezing sliding microtome. The sections were immunostained with various primary antibodies followed by confocal microscopy. PFF-488 and lysosomal marker CTSD were colocalized with various cellular markers: Astrocytes, GFAP (Agilent Technologies, Z0334); microglia, AIF1/Iba1 (FUJIFILM Wako Chemicals U.S.A., 19,741); neuron, RBFOX3/NeuN (MilliporeSigma, MAB377). PFF-488 trafficking study was done by monitoring its localization into subcellular levels (neuron, astrocytes and microglia) as well as lysosomal organelles level of brain Hippocampus area.

In this study we focused on CA1, molecular layer of dentate gyrus of hippocampus. To quantify the cell-type colocalization, 40× images were used from multiple sections from each animal. The total number of cells and colocalized puncta were counted using a HALO Quantitative Image Analyzer (RRID: SCR_018350; https://indicalab.com/halo/). Finally, the fraction of specific cellular colocalization of PFF-488 was calculated by dividing the number of PFF puncta located within the cell type marker staining (GFAP, AIF1, or RBFOX3) with the total number of the appropriate cells within the region of analysis. For each immunostaining and confococal session, multiple (4–6) serial sections (~100 μm apart) from one 3 h and one 24 h timepoint animals were analyzed. To account for session-to-session variations, all values were normalized to the average of 3 h samples for that session. Colocalization of cell-specific PFF-488 with CTSD are expressed as the Manders coefficient estimated by ImageJ software with JACop plugin (RRID: SCR_025164; https://imagej.net/plugins/jacop).

#### Analysis of lysosome function

To measure lysosomal CTSB activity in live cells, we used the Magic Red Fluorescent CTSB assay kit . Briefly, cells were plated on coverslips and following PFF-488 uptake for indicated times, the cells were washed (PBS with trypsin [0.01%]) and treated with 1x Magic Red solution for 15 min at 37°C per manufacturer instruction. Live cell confocal imaging was done immediately by confocal microscopy where the lysosomal CTSB activity is indicated by red fluorescence puncta produced by the hydrolysis of fluorogenic substrate, Magic Red. Images were taken from each culture using identical conditions and the confocal images were used to analyze the Magic Red signal in each cell using ImageJ. Briefly, each cell was outlined and the % area of the cell covered by Magic Red signal, as well as total signal intensity per cell, was determined.

To measure lysosomal membrane permeabilization, we immunostained for LGALS3/Galactin3 [31]. LGALS3 (Abcam plc, ab2785) is a sugar-binding protein that translocates from cytosol to lysosome when the membrane becomes permeable due to lysosomal damage. Once lysosome membrane permeabilization happens, the LGALS3 fluorescence pattern changes from a diffuse condition to a dotted punctate structure. For the lysosome membrane permeabilization assay, cells were plated on coverslips and treated with PFF-488 as indicated time. Cells were fixed in 4% PFA and immunostained using LGALS3 primary and AF-647 conjugated secondary antibodies. Images were taken by confocal microscopy. To measure LGALS3 accumulation in permeable lysosomes, punctate LGALS3 staining was determined using ImageJ. Briefly, each cell was outlined and the % area of the cell covered by LGALS3 immunoreactivity, as well as total signal intensity per cell, was determined.

To measure overall lysosomal function in PCN, we used the DQ-Red-BSA assay . Briefly, cells were cultured and treated with PFF-488 as mentioned above. Following indicated treatments, 10 µg/ml of DQ-BSA Red (Thermo Fisher Scientific Corporation, D12051) was dissolved in cell culture media and incubated for the last 90 min at 37°C. Fixed cells using 4% PFA at room temperature and images were taken following identical conditions throughout the treatment condition. DQ-BSA Red fluorescent signal is detected only when the fluorogenic substrate is hydrolyzed by lysosomal proteases. The red fluorescence resulting from lysosomal targeting of DQ-Red-BSA was determined using ImageJ. Because of an extensive network of neurites with DQ-Red signal in neurons, we quantified total DQ-Red signal (% area covered and total signal intensity) per microscopic field in at least 4 independent areas. The total % area covered by the DQ-Red signal was divided by the number of cells (DAPI-stained nuclei) in the field analyzed.

#### Antibodies

The primary antibodies used in this study are listed in Table 1.

**Table 1.** List of primary antibodies utilized in experiments.

Loading Controls	Host	Source	Reference	RRID	Use
GAPDH (D16H11)	Rbt	Cell Signaling Technology	5174	AB_10622025	WB
TUBA	Rbt	Abcam	4074	AB_2288001	WB
**SNCA Species**	**Host**	**Company**	**Reference**	**RRID**	**Use**
Syn-1 (total SNCA)	Rt	BD Transduction	610,787	AB_398108	WB
HsSyn	Rbt	In House	[14]	N/A	WB, ICC
LB509	Ms	Abcam	27,766	AB_727020	WB, ICC
NAC-2 and *N*-2	Rbt	Pekka Jäkälä, Kupio University	[14]	N/A	WB
Pan (Pan-SNCA/SNCB)	Rbt	Abcam	ab53726	AB_882803	WB
**Glial and Neuronal Markers**	**Host**	**Company**	**Reference**	**RRID**	**Use**
AIF1/Iba1	Rbt	Wako Chemical	019–19741	AB_839504	ICC
GFAP	Rbt	Dako Cytomation	Z0334	AB_10013382	ICC
RBFOX3/NeuN	Ms	Millipore	MAB377	AB_2313673	ICC
O4	MS	R&D System	MAB1326	AB_357617	ICC
**Autophagy/Lysosome Function/Organelle**	**Host**	**Company**	**Reference**	**RRID**	**Use**
MAP1LC3	Rbt	Cell Signaling Technology	2775, 3868	AB_915950	WB, ICC
SQSTM1/p62	Rbt	Cell Signaling Technology	5114	AB_10624872	WB, ICC
SQSTM1/p62	Ms	Abcam	ab56416	AB_945626	ICC
p-RPS6/pS6	Rbt	Cell Signaling Technology	2211	AB_331679	WB
RPS6/S6 (total)	Ms	Cell Signaling Technology	2317	AB_2238583	WB
EIF4EBP1/4EBP	Rbt	Cell Signaling Technology	9644	AB_2097841	WB
CTSD	Ms	Abcam	ab75852	AB_1523267	WB, ICC
LGALS3/Galactin3	Rbt	Abcam	ab2785	AB_2291667	ICC
Poly-UBQ	Rbt	Dako	Z0458	AB_2315524	WB
HSPA5/Grp78	Rbt	Novus	NB300-520	AB_10000968	ICC
LAMP1	Rat	LS-Bio	LS-B4246	AB_10718424	WB, ICC
LAMP2	Rbt	LS-Bio	LS-B581	AB_909691	ICC
EEA1	Rbt	Gene Tex	GTX 109,638	AB_1950162	WB, ICC
GOLGA2/GM130	Rbt	Novus	NBP2-53420	AB_2916095	ICC

Ms, mouse; Rt, rat; Rbt, rabbit; WB, western blot; ICC, immunocytochemistry.

#### Ethics statement for animal Research

All animal studies were performed in accordance with the national ethics guidelines for the use of animals in research and approved by the Institutional Animal Care and Use Committee (IACUC) at the University of Minnesota (2304-40978A, 2412-42620A).

#### Statistical analysis

To test for statistical significance between treatment groups, data was analyzed by one-way or two-way analysis of variance (ANOVA) followed by a Multiple Comparison post hoc test (Tukey’s/Dunnett’s/Bonferroni’s), or Student’s t-test. All tests were performed using GraphPad PRISM Software (Version 10; RRID:SCR_002798; https://www.graphpad.com). All the data are expressed as means ± S.E. Probability (p) values less than 0.05 were considered significantly different.

## Supplementary Material

Supplemental Material

## Data Availability

The datasets used and/or analyzed as well as a table of key resources (KRT) for the current study are available on Zenodo (10.5281/zenodo.12522122). Any additional data and materials are available from the corresponding author upon reasonable request.
