# Supplementary material for: Internalized SNCA/α-synuclein fibrils become truncated and resist degradation in neurons while glial cells rapidly degrade SNCA fibrils
Source: Autophagy. 2025 Nov 12;22(1):102–20. doi: 10.1080/15548627.2025.2579147 (PMC12758206; doi:10.1080/15548627.2025.2579147)
Supplement: Supplemental Material [file KAUP_A_2579147_SM4497.pdf]

Supplementary Figures

Figures S1-S8

Internalized SNCA/α-synuclein fibrils are rapidly truncated and resists degradation in neurons while glial cells rapidly degrade SNCA fibrils

Md. Razaul Karim, Emilie Gasparini, Elizabeth Tiegs, Riley Schlichte, Scott C. Vermilyea, and Michael K. Lee

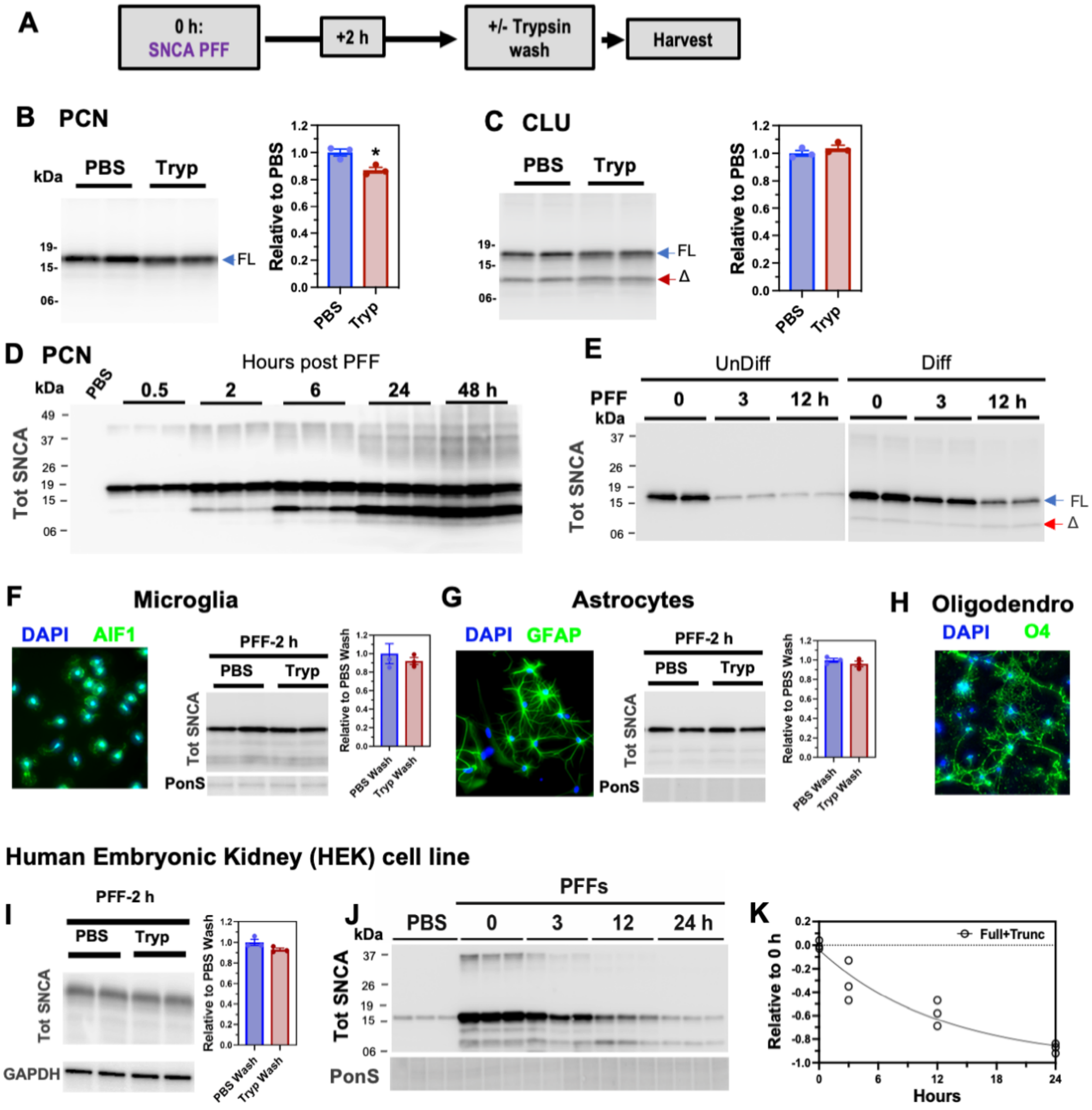

Figure S1. Uptake and metabolism of SNCA various cell types. (**A-E**) Comparison of PBS wash and trypsin wash in removing uninternalized SNCA PFF (**A-D**) and metabolism of SNCA PFF in SH-SY5Y cells (**E**). (**A**) Scheme showing cultured primary cortical neuron (**PCN, B**) or CLU198 cells (**CLU, C**) incubated with 4 µg/ml SNCA PFF for 2 h and washed with PBS or trypsin before the harvest. The levels of Tot SNCA were detected by immunoblot analysis. (**B**) In PCN, approximately ~85-90% residual SNCA following PBS wash is accounted by trypsin resistant fraction. (**C**) In CLU cells, the amount of residual SNCA is not different between PBS and trypsin-washed cells. These results indicate that the bulk of residual αS following PBS wash is internalized αS (*$p<0.05$, t-test, Mean±SEM; n=3). (**D**) Analysis of SNCA PFF uptake in PCN without washing shows that SNCA continues to increase past the 48 hours following PFF treatment. Thus, uptake of new SNCA PFF occurs for prolong periods without washing. (**E**) Uptake and metabolism of SNCA PFF in an undifferentiated (UnDiff) and neuronally differentiated (Diff) human neuroblastoma cell line (SH-SY5Y cells). Immunoblot analysis of total lysates for SNCA

shows that, similar to the CLU cells (Fig. 1G), internalized SNCA is rapidly degraded in undifferentiated SH-SY5Y cells. In differentiated SH-SH5Y cells, internalized SNCA is more stable with the presence of the truncated SNCA (Δ). (**F-I**) Uptake of SNCA PFF in glial cells and in HEK293 cells. Primary glial cells were isolated and cultured from newborn mouse pup's cortex and the purity was confirmed by staining with specific cellular marker AIF1 (**F**), GFAP (**G**), and O4 (**H**) antibodies for microglia, astrocytes, and oligodendrocytes, respectively. Microglia (**F**), astrocytes (**G**), and HEK293 (**I**) cells were also incubated with 4 μg/ml SNCA PFF for 2 h followed by PBS or trypsin wash before harvesting. The levels of Tot SNCA were detected (Mean ± SEM; n=3). (**J, K**) Degradation of SNCA PFFs in HEK-293 cells. Cells were pre-incubated for 2 h with 4 μg/ml SNCA PFF followed by trypsin wash. After washing, cells were replenished with full medium and incubated for the indicated time and levels of Tot SNCA were detected. The graph shows the amount of residual αS remaining from the 0 h time point. Ponceau S (PonS) protein stain or GAPDH were used to confirm loading.

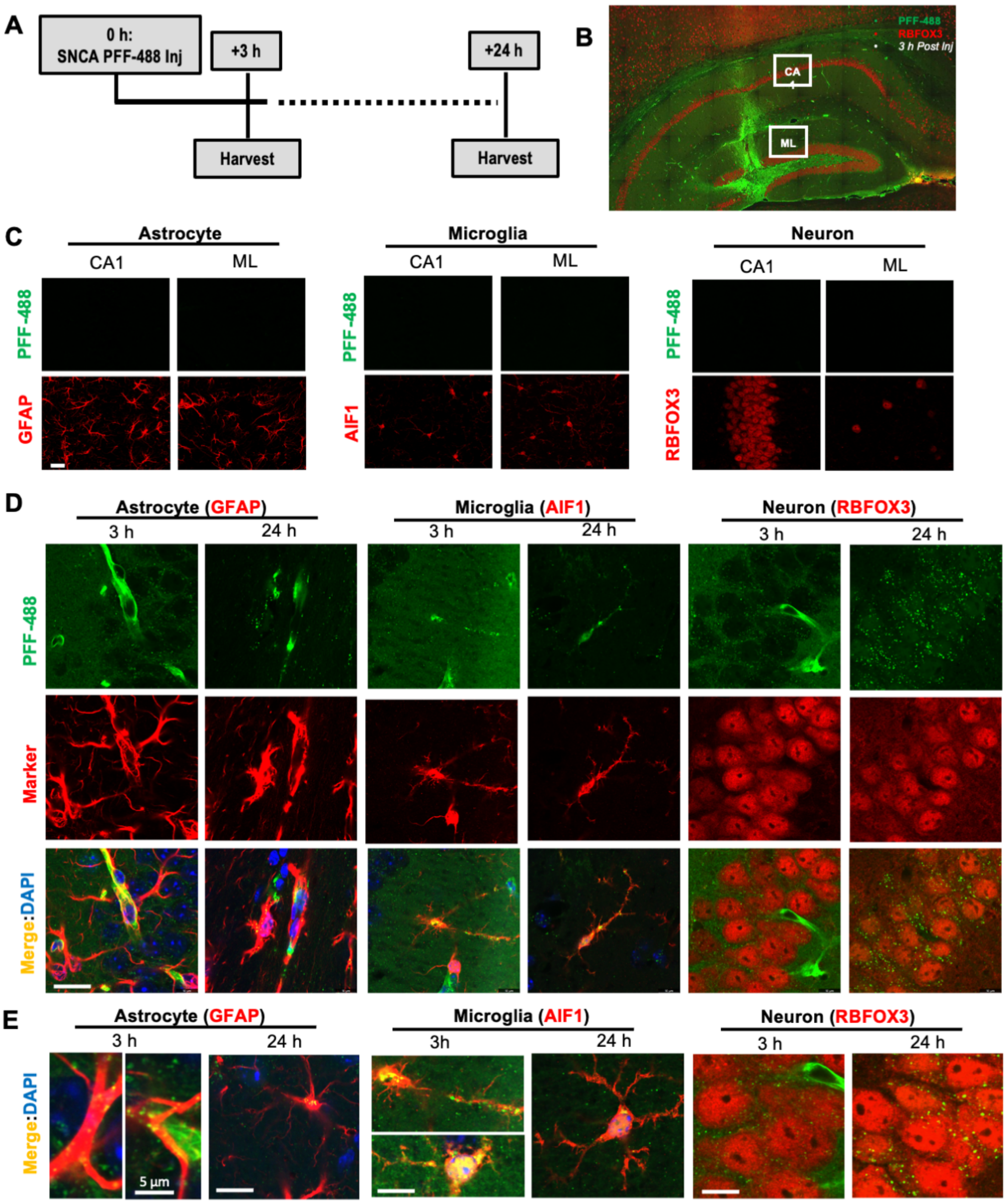

Figure S2. Cellular localization of exogenous SNCA PFF injected into mouse brain. (**A**) Schematic for the analysis of AF-488-labelled SNCA PFF (PFF-488) injected into mouse brain. (**B**) Low magnification immunofluorescence microscopic image showing PFF-488 (green) and NeuN (red) immunostaining of the brain sections taken at 3 h post injection. Note the highly levels of PFF-488 at the injection site and along the needle tract. White rectangles indicate the regions examined and quantified in **Fig. 3**. CA1-hippocampal pyramidal cell layer and ML-molecular layer dorsal to dentate gyrus. (**C**) Imaging of contralateral side for PFF-488 along with cell type markers (GFAP, AIF1, RBFOX3) at 3 h

post injection. No PFF-488 associated signal is seen. (**D**) Confocal slice image showing that the SNCA PFF-488 and the cell type markers are colocalizing within the same 1 μm plane. Bar: 20 μm. (**E**) Higher magnification images of confocal slices, similar to that shown in **D,** shows details of punctate colocalization of PFF-488 with cell-type markers within the same 1 μm plane. In astrocytes, the punctate pattern in the cell bodies at 3 h post injection are masked by the larger areas of internalized PFF-488. However, closer examination of GFAP+ glial processes clearly show punctate colocalization of PFF-488 with GFAP at 3 h post injection. AIF1+ microglia show punctate PFF-488 in both cell bodies and in the processes. Bar: 10 μm, except the 3 h GFAP panels where the Bar: 5 μm.

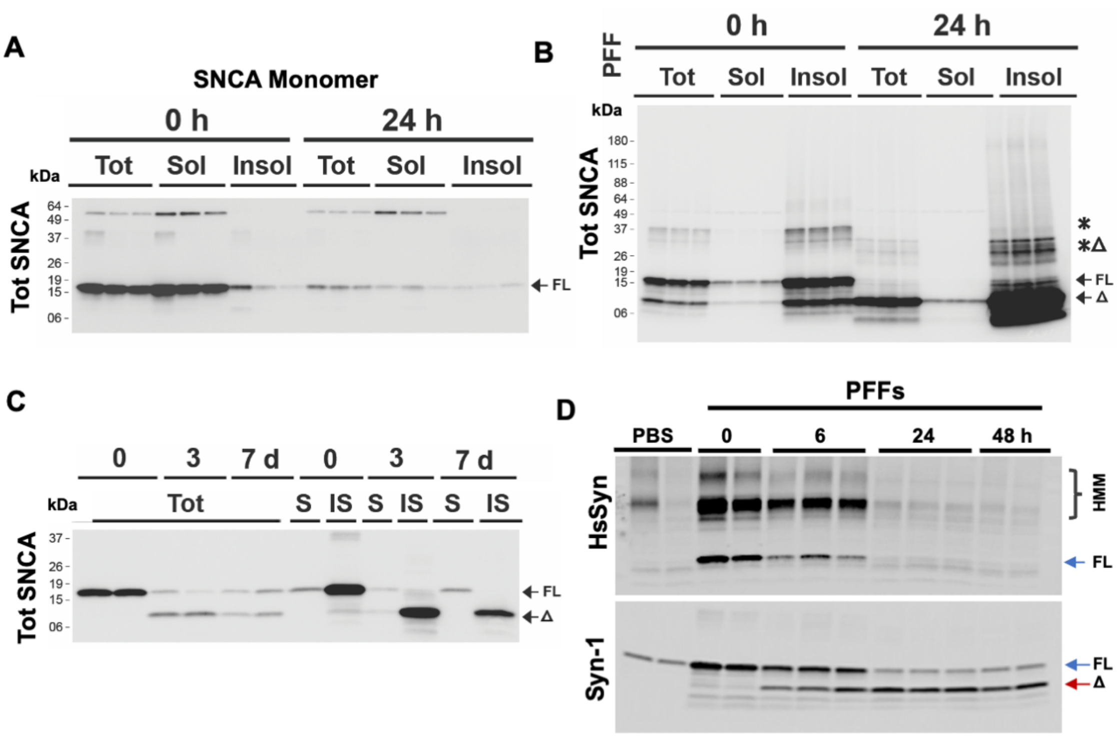

Figure S3. Internalized, truncated SNCA PFF remain detergent-insoluble. (**A, B**) Neuronally differentiated CLU198 cells were treated with 4 µg/ml of SNCA monomers (**A**) or PFF **(B)**. At 0 h and 24 h after washing, the cell lysates were fractionated into total SDS-soluble lysates (Tot) and TX-100 soluble (Sol) and insoluble (Insol) fractions. Immunoblot analysis show that internalized SNCA monomers partition with the TX-100 Sol fraction (**A**) while bulk of SNCA PFF partitions with the TX-100 Insol fraction (**B**). Note that ~37 kDa SNCA species (*) are resolving at lower MM (*Δ). (**C**) PCN treated with PFFs were analyzed for Tot SNCA in TX-100 soluble (S) and insoluble (IS) fractions at 3 d and 7 d post SNCA PFF treatment. Even at 7 d following the initial internalization of SNCA PFF, virtually all the truncated SNCA (Δ) partitions with the insoluble fraction. (**D**) Metabolism of the C-terminal SNCA epitope in neurons. Primary cortical neurons (PCN) were pre-incubated for 2 h with 4 µg/ml SNCA PFF. After washing off excess PFF, cells were replenished with full media and incubated for the indicated time. Total lysates were used for SNCA Immunoblot analysis using HsSyn (top) and Syn-1 (bottom) antibodies. Since the epitope for HsSyn antibody is located within the C-terminal region (amino acids 115-122), the results show that internalized SNCA PFF is C-terminally truncated.

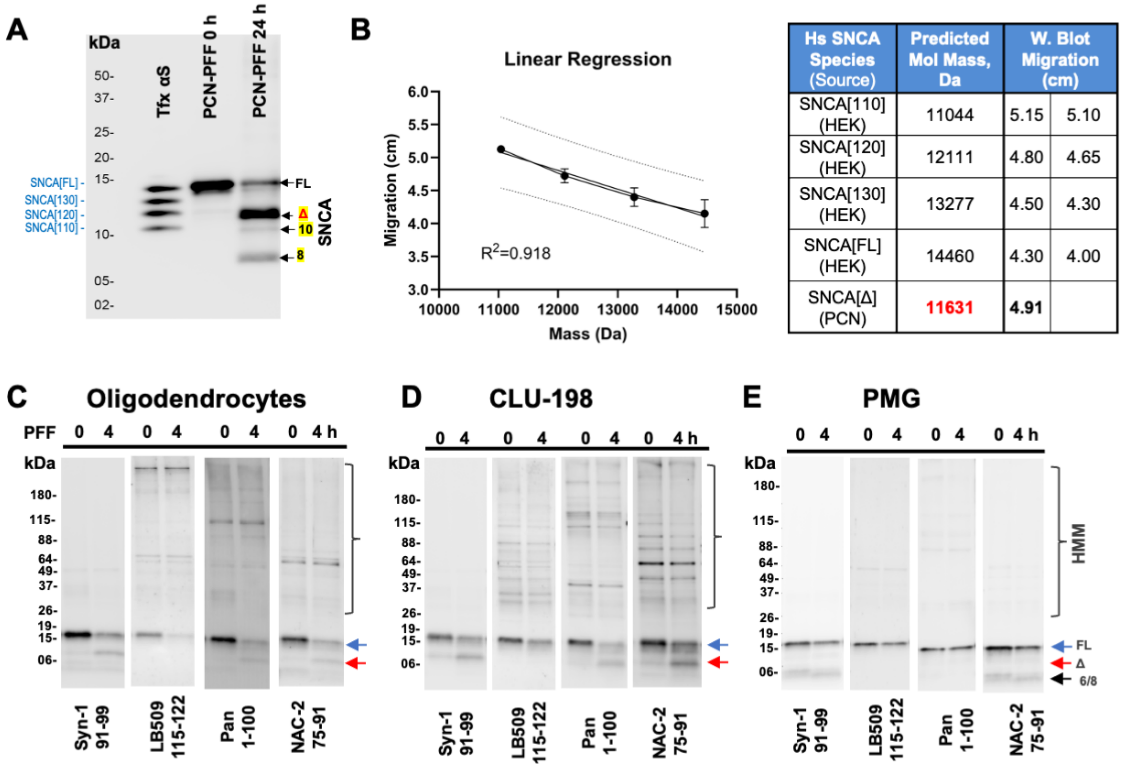

Hs SNCA Species (Source)	Predicted Mol Mass, Da	W. Blot Migration (cm)	
SNCA[110] (HEK)	11044	5.15	5.10
SNCA[120] (HEK)	12111	4.80	4.65
SNCA[130] (HEK)	13277	4.50	4.30
SNCA[FL] (HEK)	14460	4.30	4.00
SNCA[Δ] (PCN)	11631	**4.91**	

Figure S4. Molecular mass estimation of the truncated SNCA. (**A**) Total lysates from SNCA PFF treated PCN, along with cell lysates expressing full-length (FL) SNCA (SNCA[FL]) and C-terminally truncated SNCA variants (SNCA[130], SNCA[120], SNCA[110]), were resolved on a Tris-Tricine gels and immunoblotted for total SNCA. Also indicated are the major truncated SNCA(Δ) and minor 10 and 8 kDa species from internalized SNCA PFF. (**B**) Based on the relative migration of the transfected SNCA variants, the major truncated SNCA generated from SNCA PFF resolves at ~11.5 kDa. (**C-E**) Epitope mapping of internalized αS epitope in neuronal and glial cells. Primary oligodendrocytes (**C**), neuronally differentiated CLU-198 cell (**D**), and Primary microglia (PMG) (**E**) were treated with 4 µg/ml SNCA PFF and total lysates were used for epitope mapping of the truncated SNCA. In all cells, the C-terminal HsSNCA epitope recognized by LB509 antibody is missing in the truncated variants. While the N-terminal epitope recognized by the Pan antibody is present in FL and truncated (Δ) variants, the N-terminal region is missing in lower MM variant(s) (6/8).

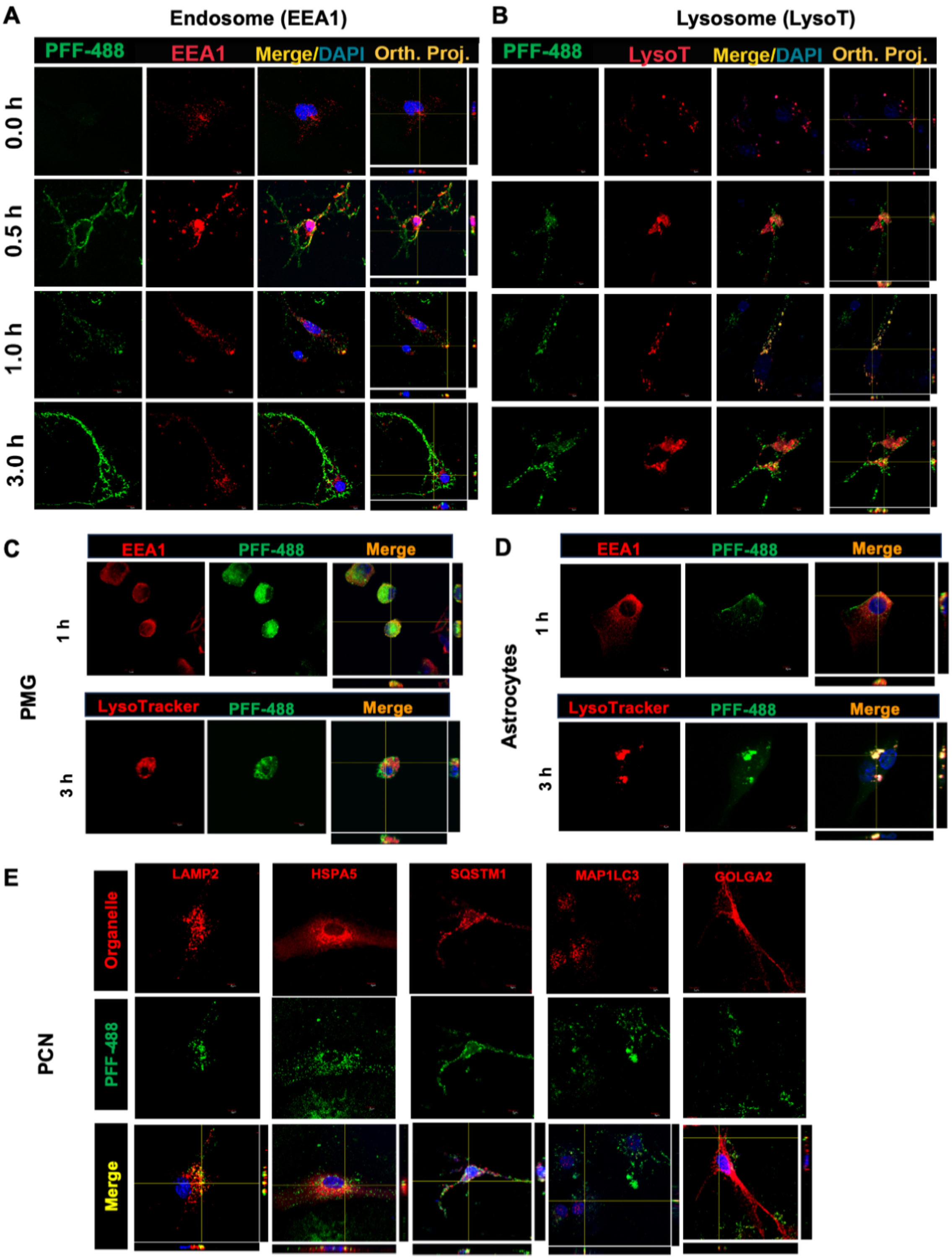

Figure S5. Cellular localization of exogenous SNCA PFF in cultured cells. (**A, B**) Individual panels associated with merged images shown in **Fig. 5A** and **5D**. PCN was treated with PFF-488 and fixed at 0, 0.5, 1 and 3 h following PFF-488 addition. Cells were immunostained for early endosome marker EEA1 **(A)** or LysoTracker Red (LysoT) (**B**).

(**C,D)** Internalized SNCA PFFs traffics to endosome and Lysosomes in glial cells. Primary microglia (PMG) (**B**) and astrocytes (**C**) were treated with PFF-488 and colocalized with the early endosome marker (EEA1) (red) at 1 h and Lysosomes (LysoTracker) at 3 h. (**E**) Localization of internalized PFF-488 with intracellular organelles. Primary cortical neurons (PCN) were treated with PFF-488 for 3 hours and the cells were washed with trypsin to remove excess PFF before fixing the cells. PFF-488 (green) were colocalized with various organelle markers: Lysosome, LAMP2; endoplasmic reticulum, HSPA5/Grp-78; lysosomal substrate, SQSTM1/p62; autophagosome, MAP1LC3; Golgi, GOLGA2/GM130.

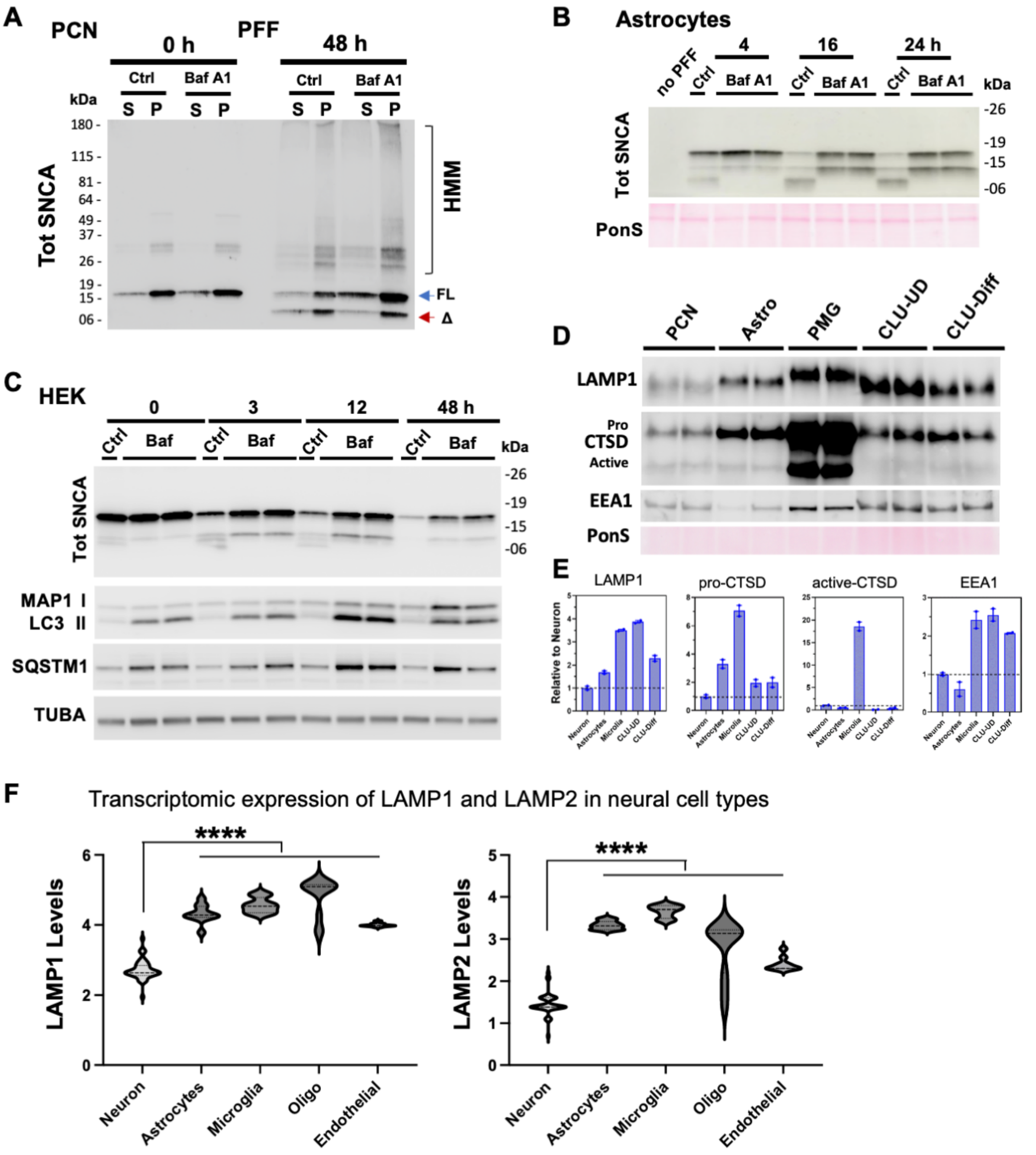

Figure S6. Lysosome is the major site of SNCA PFF metabolism. (**A**) Mouse primary cortical neurons (PCN) were pre-incubated for 2 h with 100 ηM Baf A1 prior addition of 4 µg/ml SNCA PFF for 2 h. After trypsin-wash, cells were replenished with full media and Baf A1 for the indicated time. TX-100 soluble (S) and insoluble (P) fractions were separated and analyzed for Tot SNCA (Syn-1) by immunoblotting. (**B, C**) Lysosome inhibition stabilizes internalized SNCA PFF in astrocytes (**B**) and HEK293 cells (**C**). Cells were pre-incubated for 100 ηM Baf A1 and 4 µg/ml PFF. The lysates were collected at indicated times. Immunoblot analysis of SNCA shows that while most of SNCA is metabolized by 12-16 hours in astrocytes (**B**) and HEK293 cells (**C**), Baf A1 treated cells continued accumulate of SNCA, even at 24-48 h. Also shown is Ponceau S (PonS) total protein stain or TUBA immunoblot. Immunoblot analysis of MAP1LC3-I/-II and SQSTM1

verify the inhibition of lysosomes (**C**). (**D, E**) Neuronal cells have lower lysosomal markers. (**D**) Primary cortical neuron (PCN), primary astrocytes, primary microglia (PMG), and differentiated or undifferentiated mouse hippocampal CLU cells were harvested, and lysosomal markers (LAMP1, CTSD, and EEA1) were evaluated by immunoblotting. **E)** Quantitative analysis of immunoblot shown in **D**. While statistical analysis was not done, the duplicate samples clearly show that lysosomal markers are lower in neurons than in non-neuronal cells. **F)** Whole brain cell type expression levels were extracted for Neurons, Astrocytes, Microglia, Oligodendrocytes, and Endothelial cells from DropViz single cell transcriptomic data base for LAMP1 (http://dropviz.org/?_state_id_=c8c0e49d46a99f4b) and LAMP2 (http://dropviz.org/?_state_id_=89519e3741f26c75). The violin plot of the values show that neurons express less LAMP1 and LAMP2 than other brain cell types. ****$p<0.0001$, *One Way ANOVA.*

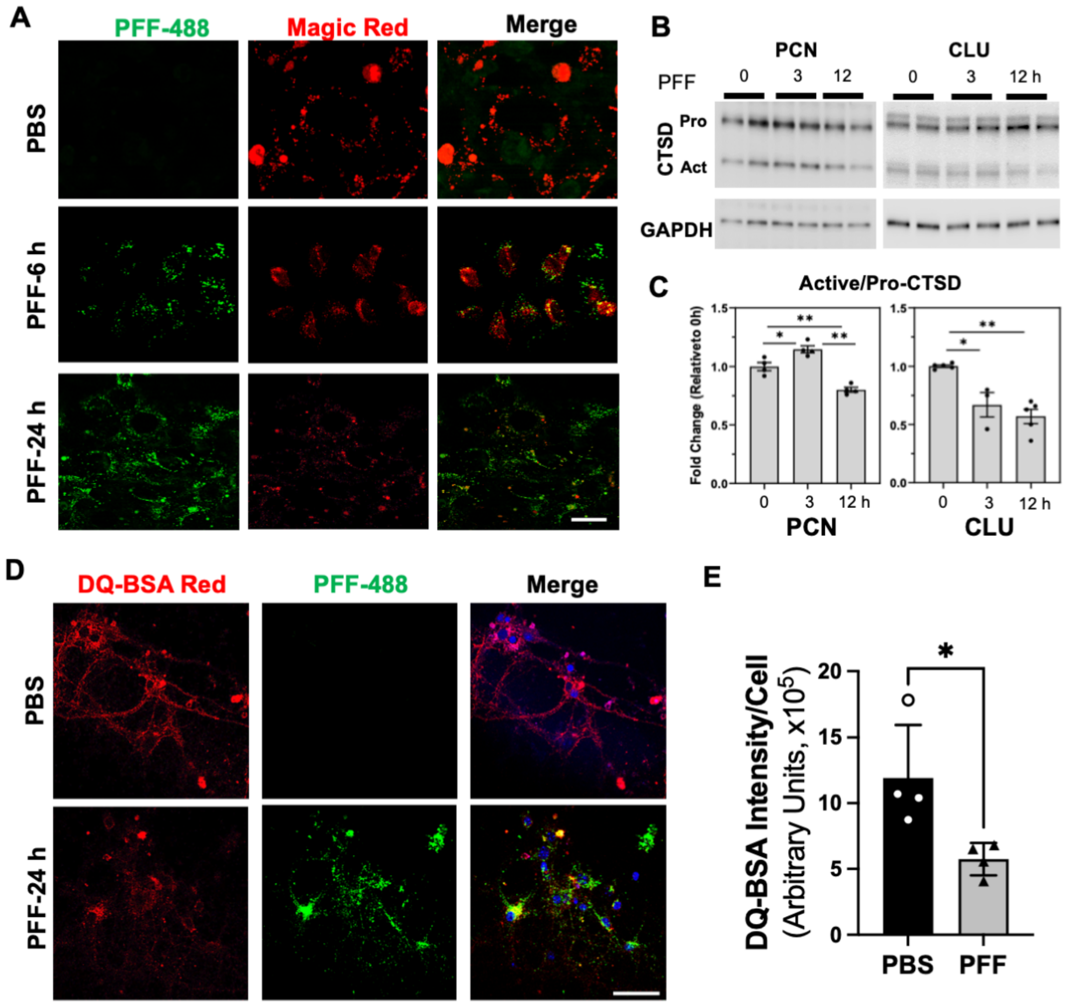

Figure S7. Internalized SNCA PFF inhibit lysosomal function in neuronal cells. (**A**) Differentiated CLU198 cells were treated with PFF-488, washed, and imaged at 6 or 24 h for PFF-488 or Magic Red. The Magic Red signal is reduced in PFF-treated cells. Quantitative analyses of 24 h time point is shown in **Fig. 7**. Bar: 10 µm. (**B**) PFF treated PCN or CLU198 cells were analyzed for the relative amount of active- and pro-CTSD) by immunoblotting. (**C**) Immunoblot shown in **B** were quantified, showing that the PFF treatment reduced the relative levels of active-CTSD, relative to the pro-CTSD. *$p<0.05$, **$p<0.01$, One-Way Anova, Tukey's multiple comparison test. (**D, E**) Primary cortical neurons were treated with PFF-488, washed, and incubated for 24 h and treated with DQ-BSA Red, which is hydrolyzed in functional lysosomes to produce red fluorescence. Confocal images show that PFF treatment is associated with reduced DQ-BSA Red signal (**D**). Quantitative analysis of total DQ-BSA Red signal intensity, normalized to the number of cells, confirms that PFF treatment leads to lysosomal dysfunction in neurons (**E**). *$p<0.05$, unpaired *t*-test, n=4 independent cultures. Bar: 50 µm (C). *$p<0.05$, unpaired t-test.

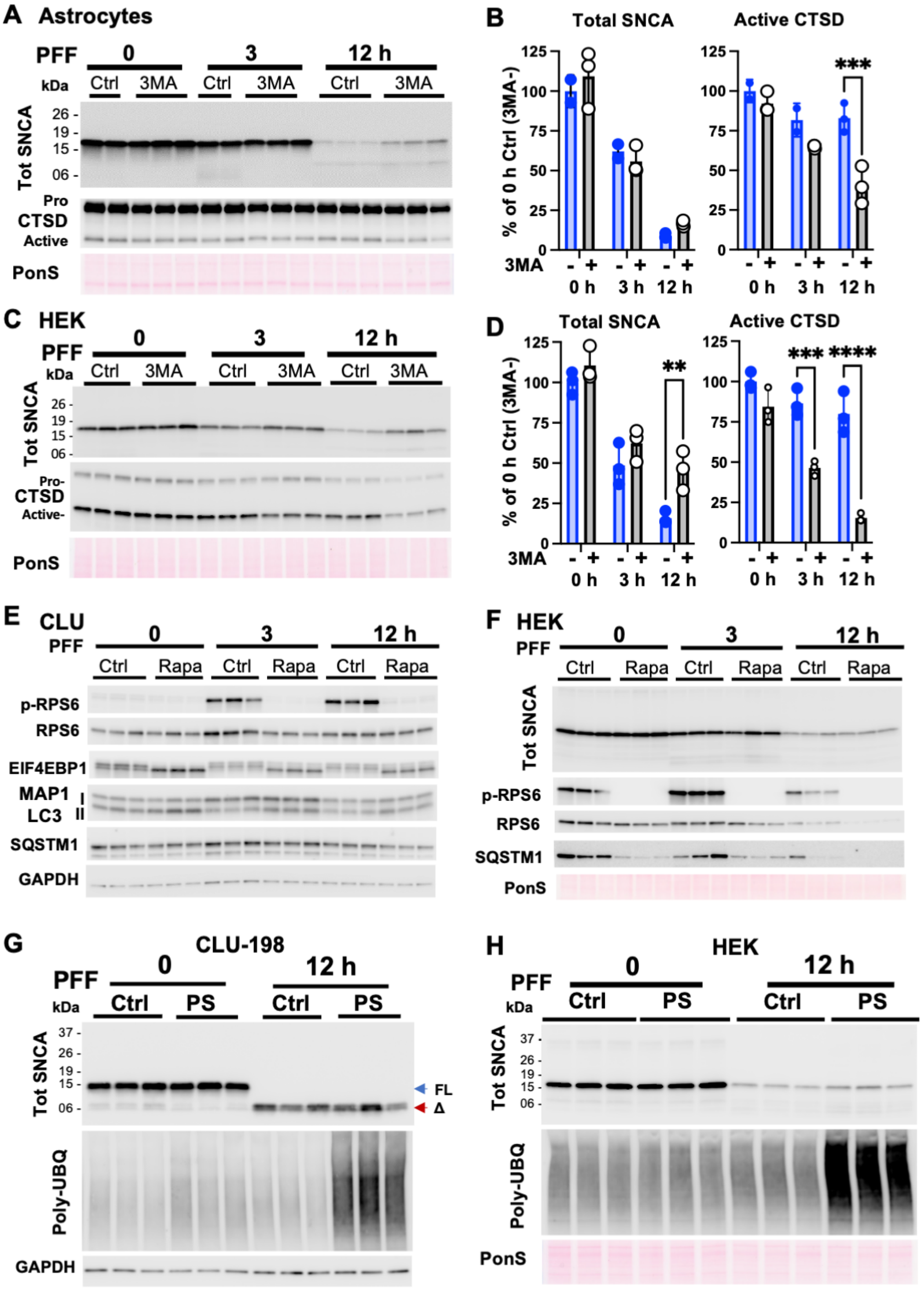

Figure S8. Autophagy and proteasome do not have an impact upon SNCA PFF metabolism. (**A-D**) Inhibition of autophagy leads to modest lysosomal inhibition with a

modest impact on SNCA accumulation in astrocytes and HEK293 cells. Primary cultures of astrocytes (**A, B**) and HEK293 cell line (**C, D**) treated with 3MA to inhibit autophagy. The levels of internalized SNCA PFF and the lysosomal protease, CTSD, determined by immunoblot analysis, show that 3MA treatment modestly but significantly increased SNCA levels in HEK293 cells (**C, D**). 3MA also decreased active-CTSD levels in both Astrocytes (**B**) and HEK293 (**D**) cells. ***p<0.01, ***p<0.001, ****p<0.0001*, Two-way ANOVA. (**E, F**) Rapamycin (Rapa) increases autophagy but does not affect SNCA PFF metabolism. (**E**) CLU-198 shown in **Fig. 8D** analyzed for MTOR inhibition (p-RPS6, EIF4EBP1) and autophagy (MAP1LC3, SQSTM1). In addition to inhibition of MTOR, Rapa treatment leads to increased MAP1LC3-II and reduced SQSTM1, indicating to enhanced autophagy. (**F)** In HEK293 cells, Rapa treatment does not affect the metabolism of internalized SNCA. (**G, H**) Proteasome inhibition does not have an impact upon SNCA PFF processing/metabolism. (**G**) Differentiated mouse hippocampal cell CLU-198 and (**H**) HEK-293 cells were pre-incubated with 15 ηM PS-341 (PS; 4 h) and 4 µg/ml SNCA PFF for 2 h before the trypsin-wash. After the trypsin wash, cells were replenished with full media and PS for the indicated time. Tot SNCA, poly-ubiquitin (poly-UBQ) were detected by immunoblotting analysis (**G, H**). GAPDH (**G**) or Ponceau S (PonS)(**H**) used as loading controls. PS-341 treatment does not have an impact upon SNCA truncation or metabolism in CLU-198 cells (**G**) and does not have an impact upon SNCA metabolism in HEK293 cells (**H**).
